# ASL perfusion radiomic heterogeneity is associated with early mortality in IDH-wildtype glioblastoma, beyond tumor volume: a time-varying effect with tumor-aware DTI-ALPS

**DOI:** 10.3389/fonc.2026.1918963

**Published:** 2026-09-09

**Authors:** Rafail C. Christodoulou, Georgios Vamvouras, Platon S. Papageorgiou, Evros Vassiliou, Elena E. Solomou, Sokratis G. Papageorgiou, Michalis F. Georgiou

**Affiliations:** 1 Division of Neuroimaging and Neurointervention, Department of Radiology, Stanford University, Stanford, CA, United States; 2 Department of Electrical and Computer Engineering, National Technical University of Athens (NTUA), Athens, Greece; 3 Department of Medicine, National and Kapodistrian University of Athens, Athens, Greece; 4 Department of Biological Sciences, Kean University, Union, NJ, United States; 5 Internal Medicine-Hematology, University of Patras Medical School, Rion, Greece; 6 1st Department of Neurology, Medical School, National and Kapodistrian University of Athens, Eginition Hospital, Athens, Greece; 7 Department of Radiology, University of Miami, Miami, FL, United States

**Keywords:** arterial spin labeling (ASL), explainable AI, glioblastoma, glymphatic system (GS), mortality prediction

## Abstract

**Background:**

Glioblastoma exhibits significant spatial heterogeneity, with perfusion variability that may reflect angiogenesis, hypoxia, and biological aggressiveness. Arterial spin labeling (ASL) offers non-contrast perfusion imaging, and radiomics quantifies tumor texture beyond basic ROI metrics. However, radiomic texture features can be confounded by tumor volume -- rarely tested directly. Diffusion tensor imaging along the perivascular space (DTI-ALPS) may capture perivascular/neurofluid dynamics, but its added prognostic value in glioblastoma is uncertain.

**Methods:**

We retrospectively included 322 patients with IDH-wildtype WHO grade 4 glioblastoma. ASL radiomic features (shape features excluded) were tested against tumor volume, clinical covariates, and DTI-ALPS in volume-adjusted Cox proportional hazards models. A machine-learning classification analysis of 12-month mortality (N=259; ALPS-valid subset N=100) compared clinical, volume-aware, and ASL-augmented models across six classifiers, with proportional-hazards assumptions formally tested.

**Results:**

Higher ASL heterogeneity was associated with mortality within 365 days of imaging, after adjustment for tumor volume and clinical variables (HR 1.39, 95% CI 1.07 -1.81, p=0.015), with proportional hazards confirmed within this window. This association persisted when the candidate feature pool was widened from the pre-specified 26-feature family to all 1,209 non-shape ASL features screened within training folds (HR 1.33, 95% CI 1.05 -1.69). The association was unchanged under flexible modeling of tumor volume (HR 1.40 -1.49 across spline, polynomial, and quantile-indicator specifications), showed no heterogeneity across volume strata (I²=0%), and persisted after orthogonalizing the score with respect to volume (HR 1.25 -1.27, all p<0.03). The association was time-varying -- strongest in the first 180 days (HR 1.37) and attenuating beyond one year. In contrast, the unadjusted association with overall survival did not remain significant after accounting for tumor volume, and machine-learning classification showed no incremental value from ASL radiomics beyond a volume-aware clinical baseline. DTI-ALPS did not improve classifier performance or show independent survival association.

**Conclusions:**

ASL perfusion heterogeneity was associated with early, but not overall, mortality in this cohort, with a modest component not explained by tumor volume -- a signal specific to the first year after imaging. Findings for overall survival and machine-learning classification were substantially attributable to tumor volume rather than radiomic texture, underscoring the importance of volume-adjusted testing in radiomics research. Tumor-aware DTI-ALPS provided no additional prognostic value. These findings are hypothesis-generating and require prospective, multi-center validation.

## Graphical Abstract

**Figure:**
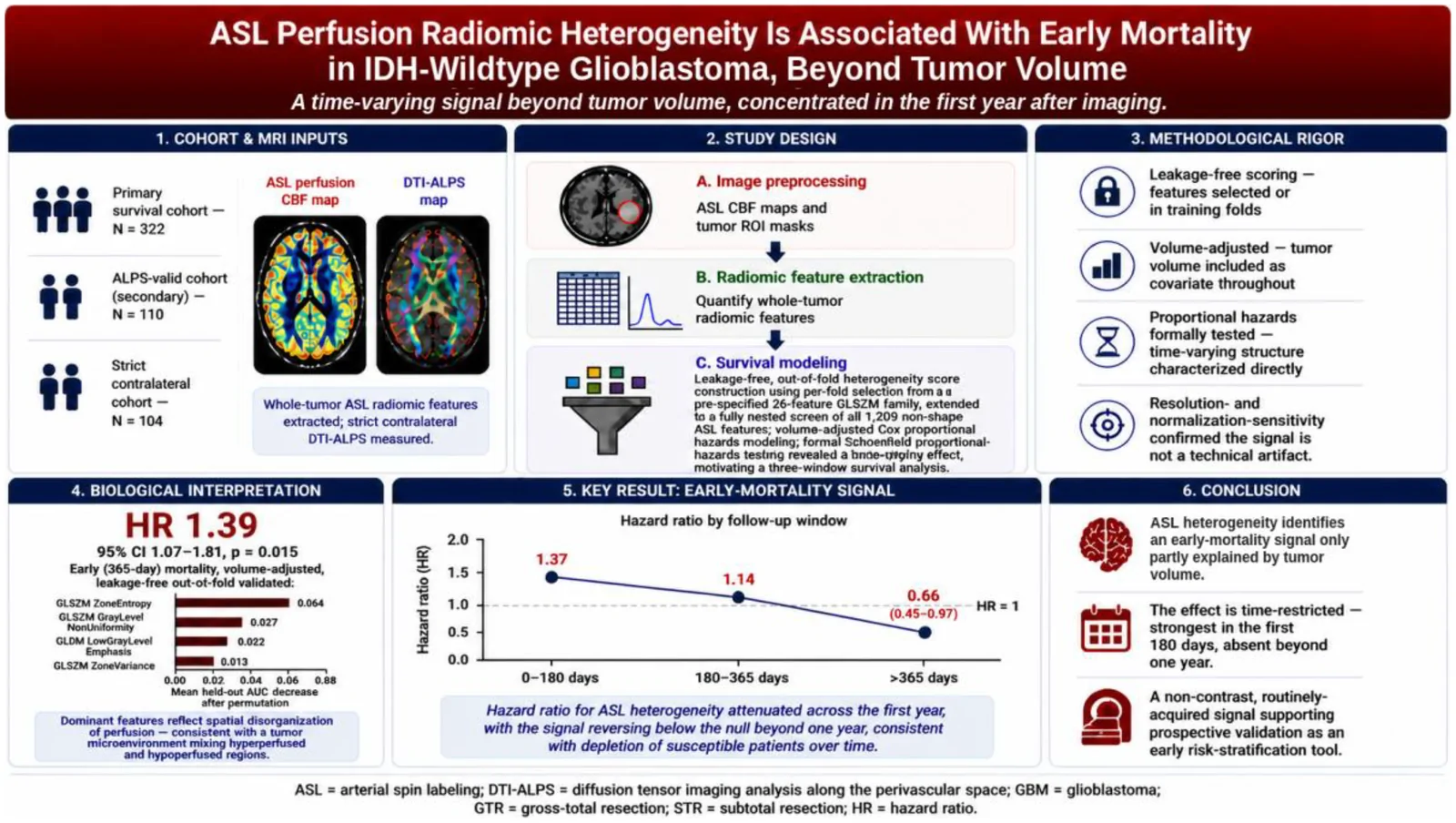

## Introduction

Glioblastoma is the most aggressive primary brain tumor in adults, constituting about 50% of adult glioma cases (1). Even though extensive research is ongoing to improve our understanding of glioblastoma’s biology and treatment response, overall survival rarely exceeds 15 months (2), highlighting the urgent need for non-invasive biomarkers to support early risk stratification and guide patient management. Perfusion MRI is useful clinically as it provides information about the tumor’s neoangiogenesis, hypoxia, and biological aggressiveness. Arterial spin labeling(ASL) provides a non-contrast measure of cerebral blood flow, has shown prognostic value in gliomas, and is associated with progression-free and overall survival (3, 4). In parallel, recent studies have suggested that perivascular fluid and glymphatic-related MRI markers, including diffusion tensor imaging analysis along the perivascular space (DTI-ALPS), reflect tumor-related disruption of neurofluid dynamics and may have prognostic relevance in glioblastoma (5).

Glioblastoma exhibits spatial heterogeneity, making simple region-of-interest perfusion measurements insufficient to capture the tumor’s vascular complexity. Similar issues have been noted with traditional perfusion methods that focus on focal hot-spot measurements representing an area of focal increased blood flow on MRI, which often underestimate the total lesion volume and intratumoral heterogeneity (6, 7). As a result, whole-tumor approaches are essential to better quantify perfusion patterns that could more accurately indicate tumor aggressiveness and survival risk.

Radiomics offers a solution to this limitation by extracting high-dimensional quantitative features from medical images, including intensity, texture, and spatial distribution characteristics (8–10). These features can capture tumor heterogeneity across the entire image and provide valuable insights into information that may not be apparent to radiology readers. In brain tumor imaging, radiomics have been applied to glioma grading, molecular prediction, treatment-response assessment, and survival prognostication (11, 12). Previous research indicates that diffusion- and perfusion-weighted MRI radiomics enhance prognostic models for glioblastoma, with perfusion-derived features potentially reflecting vascular and microstructural tumor characteristics that are important for predicting survival outcomes (13, 14).

Despite recent advances, radiomic texture features can be influenced by tumor volume, even when explicit shape features are not included, because several texture descriptors are mathematically affected by lesion size. However, this confounding factor has rarely been directly examined in perfusion radiomics studies of glioblastoma. It is also still unclear whether tumor-aware DTI-ALPS provides prognostic value that is independent of or adds to ASL radiomics. We hypothesized that heterogeneity in ASL radiomics would correlate with mortality beyond tumor volume and clinical factors, and that tumor-aware DTI-ALPS would offer complementary prognostic information as a secondary biomarker. To test this, we employed fully nested, leakage-free out-of-fold feature selection and volume-adjusted survival modeling, with proportional-hazards assumptions and time-varying effects formally assessed, considering the potential for non-constant hazard ratios over follow-up. ASL radiomics served as the main imaging biomarker, with machine-learning classification and exploratory imaging-phenotype stratification included as secondary analyses alongside the primary volume-adjusted survival analysis.

## Materials and methods

### Study design and dataset

**Figure 1:**
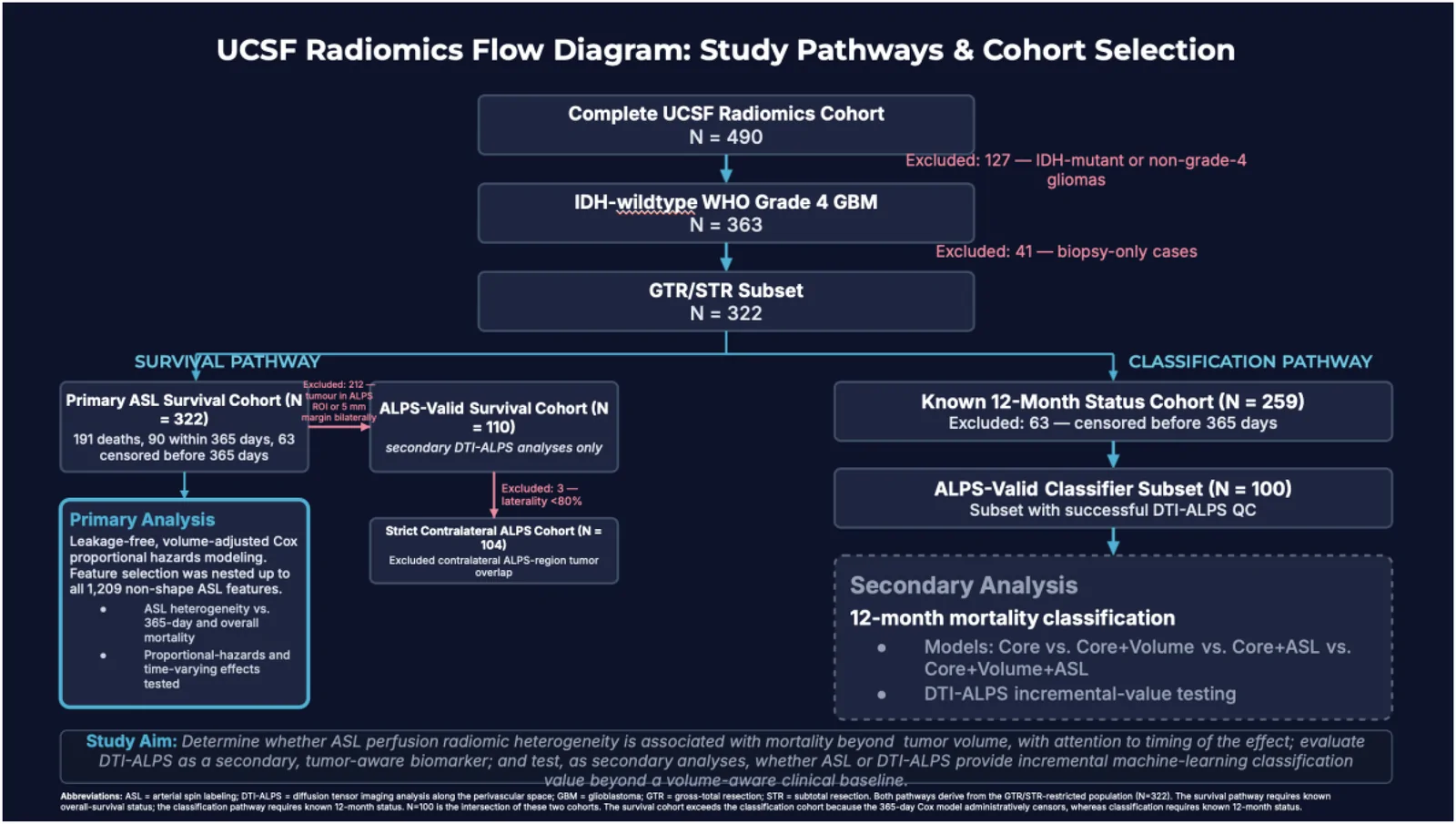
CONSORT-style cohort flow diagram showing enrollment, exclusions, and censoring status at each step.

This retrospective imaging-biomarker and machine-learning study used the University of California, San Francisco Preoperative Diffuse Glioma MRI TCIA dataset (UCSF-PDGM) (15), to evaluate prognostic information from ASL perfusion radiomics and diffusion tensor imaging analyses along the perivascular space in glioblastoma. ASL radiomics was evaluated as the primary imaging biomarker, whereas DTI-ALPS was evaluated as a secondary, tumor-aware biomarker to test whether a complementary perivascular diffusion phenotype provided independent or incremental prognostic value. The primary objective was to determine whether ASL-derived radiomic heterogeneity was associated with mortality, after adjustment for tumor volume and clinical variables, in patients with IDH-wildtype WHO grade 4 glioblastoma, using fully nested, leakage-free out-of-fold feature selection and volume-adjusted Cox proportional hazards modeling. Because proportional-hazards assumptions could not be made *a priori*, time-varying effects were also formally evaluated. As a secondary analysis, binary machine-learning prediction of death within 12 months after imaging was evaluated to test whether ASL radiomics or DTI-ALPS improved discrimination beyond a volume-aware clinical baseline. The secondary objective was to determine whether DTI-ALPS was independently associated with survival or improved prediction beyond clinical variables and ASL radiomics. The overall study workflow, including cohort assembly, imaging-biomarker extraction, machine-learning classification, and survival modeling, is summarized in **Figure 1**.

### Cohort assembly and endpoint definitions

Patients were selected from a complete, clinically matched radiomics cohort of 490 cases. The main disease-specific population consisted of 363 patients with IDH-wildtype WHO grade 4 glioblastoma. Of these, 322 underwent gross-total or subtotal resection and constituted the analytic cohort used throughout this study; the remaining 41 biopsy-only cases were excluded. IDH-mutant gliomas were excluded because they represent a biologically distinct entity with substantially longer survival, which would confound prognostic modeling (16). Biopsy-only cases were also excluded from the analytic cohort because the extent of resection is strongly associated with survival in glioblastoma, and biopsy-only patients often represent a clinically different subgroup with more limited surgical treatment, different tumor accessibility, or worse baseline prognosis (17). This GTR/STR restriction was applied uniformly to all analyses, including the ASL radiomics, DTI-ALPS, and survival cohorts described below.

For machine-learning classification, patients were included if their 12-month survival status was known. The primary ASL classifier cohort included 259 patients, of whom 90 died within 365 days after imaging and 169 were alive at or beyond 365 days. Patients censored before 365 days were excluded from the 12-month classification endpoint.

Separate ALPS-valid cohorts were assembled for analyses requiring successful DTI-ALPS processing and quality control, restricted to GTR/STR cases consistent with the rest of the analytic cohort. The primary Cox proportional hazards analysis of ASL heterogeneity was performed in the full GTR/STR cohort (N = 322, 191 deaths, 90 within 365 days), which does not require a valid DTI-ALPS measurement. Because DTI-ALPS is a secondary biomarker in this study, conditioning the primary ASL analysis on successful ALPS quality control would have excluded eligible patients for no analytic reason. Sixty-three patients in this cohort were censored before 365 days; they contribute follow-up and are administratively censored in the 365-day Cox model, which is why the survival cohort is larger than the classification cohort, the latter requiring known 12-month status. The ALPS-valid survival cohort (N = 110) was used only for analyses requiring an ALPS measurement: the low-ALPS covariate, the heterogeneity by low-ALPS interaction, and the contralateral sensitivity analysis. The ALPS-valid classifier cohort (N = 100) was used to test, as a secondary analysis, whether ALPS or ASL radiomics improved 12-month mortality prediction beyond a volume-aware clinical baseline. A strict contralateral ALPS cohort (N = 104) was also assembled for tumor-side-aware sensitivity analysis. Cohort assembly, endpoint definitions, censoring status, and the analytic role of each cohort are shown in **Table 1**. Baseline characteristics of patients included versus excluded at each major exclusion step are reported in **Supplementary Table 8**.

**Table 1:** Cohort assembly and endpoint definitions.

Cohort	N	Deaths (Events)	Median OS (days)	Deaths ≤12 Months	Alive ≥365 Days	Censored <365 Days	Purpose
Complete UCSF radiomics cohort	490	243	421	115	275	100	All clinical and radiomics cases
IDH-wildtype WHO grade 4 GBM	363	219	358	112	176	75	Disease-specific cohort
IDH-wildtype grade 4 GBM with GTR/STR	322	191	387	90	169	63	Primary ASL survival cohort (primary Cox analysis)
IDH-wildtype grade 4 GBM with GTR/STR and known 12-month status	259	191	483	90	169	0	Primary 12-month mortality classification cohort
IDH-wildtype grade 4 GBM with GTR/STR and valid ALPS	**110**	**79**	**426**	**36**	**64**	**10**	ALPS-valid survival cohort (secondary ALPS analyses only)
IDH-wildtype grade 4 GBM with GTR/STR, valid ALPS, and known 12-month status	**100**	**79**	**464**	**36**	**64**	**0**	Final ALPS classification cohort

OS, overall survival; GBM, glioblastoma; GTR, gross-total resection; STR, subtotal resection; ALPS, analysis along the perivascular space

Deaths (events) represent observed deaths during follow-up and were treated as events in survival analyses. Twelve-month deaths were defined as deaths occurring within 365 days after the baseline imaging examination. Patients alive with at least 365 days of follow-up constituted the negative class for 12-month mortality prediction. Patients who were alive but had fewer than 365 days of follow-up were considered censored before 365 days and were excluded from classification analyses because their 12-month survival status could not be determined. DTI-ALPS was computed using a single FSL-based pipeline applied uniformly across all cohorts. Consequently, the ALPS survival cohort (N = 110) includes 10 patients who were censored before 365 days, whereas the final ALPS classification cohort (N = 100) excludes these individuals. This explains why both cohorts contain the same numbers of deaths (79) and 12-month deaths (36), yet differ in sample size and median overall survival (426 vs. 464 days).

**Table 1**. Cohort assembly and endpoint definitions.

### Image acquisition and tumor segmentation

All imaging in the UCSF-PDGM dataset was acquired on a 3.0 Tesla scanner (Discovery 750, GE Healthcare, Waukesha, Wisconsin, USA) using a dedicated 8-channel head coil (Invivo, Gainesville, Florida, USA), as part of a standardized preoperative brain tumor protocol. The protocol included 3D T2-weighted, T2/FLAIR-weighted, susceptibility-weighted, diffusion-weighted, pre- and post-contrast T1-weighted, 3D arterial spin labeling perfusion, and high-angular-resolution diffusion imaging sequences. Detailed ASL-specific acquisition parameters (labeling scheme, post-label delay, and whether reported values represent absolute or relative cerebral blood flow) beyond this general protocol description are not available in the source dataset documentation.

Tumor segmentation was performed as part of the construction of the UCSF-PDGM dataset and was not independently repeated in this study. Images first underwent automated segmentation using an ensemble of prior BraTS-challenge-winning algorithms, followed by manual correction by trained radiologists. Segmentation comprised three compartments (enhancing tumor, non-enhancing/necrotic tumor, and surrounding FLAIR abnormality/edema), which were binarized into a whole-tumor mask for radiomic feature extraction as described below. A quantitative inter-rater reliability statistic for this segmentation was not reported in the source dataset documentation and was not independently assessed in this study.

### Clinical model

The core clinical model included age at MRI, sex, MGMT promoter methylation status, and extent of resection. Variables were encoded as age at MRI, male sex, MGMT-positive status, MGMT negative, and gross-total resection. This clinical model served as the baseline model against which ASL radiomics and DTI-ALPS were tested. Given the volume-sensitivity of several radiomic texture features, total tumor volume was quantified from the segmentation mask and, after natural-logarithm transformation, evaluated as an additional covariate in an extended clinical model (core clinical variables plus log-transformed tumor volume), used throughout the survival and classification analyses to test whether ASL radiomics or DTI-ALPS added incremental prognostic or discriminative value beyond tumor size. The clinical model, including extent of resection, reflects a postoperative risk-stratification context; this study does not propose a preoperative predictive use-case, and extent of resection is available at the time all imaging-based predictions in this study would be applied.

### ASL radiomics feature extraction

ASL radiomic features were extracted from the tumor segmentation mask. Feature families included first-order features, gray-level co-occurrence matrix features, gray-level run-length matrix features, gray-level size-zone matrix features, gray-level dependence matrix features, and neighboring gray-tone difference matrix features. Shape features were excluded to prioritize analyzing ASL perfusion intensity and texture over tumor size or segmentation geometry. From an initial set of 1,223 ASL features, 14 shape features were removed, yielding 1,209 non-shape ASL radiomic features. However, excluding explicit shape features does not guarantee independence from tumor volume as several non-normalized texture descriptors, including gray-level non-uniformity measures and z-scored intensity-energy features, are mathematically sensitive to voxel or zone count and can scale with lesion size even without an explicit shape term. We therefore quantified total tumor volume directly from the segmentation mask and treated its natural logarithm as the adjustment covariate in the survival and classification analyses, rather than relying solely on shape-feature exclusion to control for tumor size (18).

### ASL radiomics preprocessing and feature selection

Preprocessing for ASL radiomics extraction proceeded as follows: the UCSF-PDGM-provided ASL NIfTI volume and tumor segmentation mask were used in their native co-registered dataset space; per the UCSF-PDGM dataset description, all sequences, including ASL, were co-registered to a common 1 mm isotropic T2/FLAIR-defined grid using non-linear registration prior to release, and native-resolution acquisitions are not available in this release. No additional affine or deformable registration was estimated by our pipeline beyond this dataset-level co-registration. Before feature extraction, the tumor segmentation was converted to a binary whole-tumor mask using all positive segmentation labels. If needed, the mask was resampled to 1 × 1 × 1 mm isotropic spacing using nearest-neighbor interpolation to preserve label integrity, and the ASL image was resampled onto the final mask grid, matching image size, spacing, origin, and direction, using B-spline interpolation, consistent with the dataset’s existing 1 mm grid. To assess whether fine-scale texture attributable to this dataset-level interpolation could be driving the heterogeneity signal, we performed a resolution-sensitivity analysis in which ASL images were coarsened to approximately 3 mm (typical of native pseudo-continuous ASL acquisition) before re-extraction; the resulting heterogeneity score correlated strongly with the standard-resolution score (r = 0.94) and preserved the volume-adjusted 365-day association (hazard ratio 2.68 at coarse resolution versus 2.22 at standard resolution), indicating the signal is not attributable to interpolation-manufactured texture; this preprocessing sensitivity analysis was performed in the ALPS-valid subset (N = 110), the cohort for which coarse-resampled and non-normalized features were extracted, and is reported on that cohort throughout (full comparison in **Supplementary Table 7**). These are subset-specific sensitivity estimates in a smaller, tumor-location-selected subgroup (N = 110, 36 deaths within 365 days) and are not directly comparable with the primary N = 322 estimate of 1.39; their confidence intervals overlap the primary estimate, and the larger point estimates reflect the restricted subgroup and its reduced precision rather than a different headline effect size.

ASL images were not N4 bias-field corrected because ASL is a perfusion-derived quantitative sequence rather than a structural sequence subject to the same bias-field assumptions. Intensities were z-scored within the tumor mask before radiomic feature extraction. This normalization is standard practice for ASL radiomics, as absolute cerebral blood flow values are influenced by scanner calibration, labeling efficiency, arterial transit time, and systemic hemodynamic factors unrelated to tumor biology, limiting cross-patient comparability of unnormalized perfusion intensity. Accordingly, the ASL heterogeneity score characterizes relative perfusion heterogeneity across the composite whole-tumor mask, which comprises enhancing tumor, non-enhancing/necrotic tumor, and surrounding FLAIR abnormality/edema, rather than absolute perfusion magnitude; in a sensitivity analysis using non-normalized, absolute-CBF-derived features, the volume-adjusted early-mortality association was not observed (**Supplementary Table 7**), consistent with normalization appropriately isolating prognostically relevant relative heterogeneity from inter-patient physiological and technical variability. Feature-selection steps for the machine-learning classification analysis were performed within the training folds to reduce information leakage. Missing values were imputed using training-fold medians, and features were standardized using training-fold means and standard deviations. Near-zero-variance features were removed. Remaining features were ranked by ANOVA F-test association with the 12-month mortality endpoint, with ties ranked by absolute univariate AUC distance from 0.5. The top 80-ranked features underwent correlation-based redundancy pruning, in which highly correlated features were removed using a Pearson correlation threshold of |r| ≥ 0.90. After pruning, the final fold-specific ASL feature set was capped at 12 features in the main classifier cohort and 8 features in the smaller ALPS-valid classifier cohort to prevent overfitting (per-fold selected feature frequencies reported in **Supplementary Table 9**). The ASL heterogeneity score used for the primary survival analysis was constructed using a related but distinct leakage-free, out-of-fold procedure, described in Survival analysis.

### DTI-ALPS processing and tumor-aware quality control

DTI-ALPS was calculated using an FSL-based pipeline as described (19). Side-specific ALPS values were obtained from both hemispheres. The bilateral mean ALPS, obtained by averaging the left and right ALPS, served as an initial global measure. Because ALPS is derived from diffusivities in projection and association fiber regions near the lateral ventricles, measurements may be affected by tumor infiltration and edema. To address this, tumor-aware quality control was performed on the GTR/STR-restricted analytic population (N = 322): all 322 patients had DTI acquired and met tensor-fitting eligibility criteria (4D diffusion data present with correctly formatted rotated b-vectors), and the FSL pipeline succeeded on all attempted cases. However, 212 patients were excluded because the tumor extended into an ALPS region of interest or its 5-mm safety margin bilaterally (163 with direct tumor-ROI overlap, 49 within the safety margin only), yielding a bilateral ALPS-valid survival cohort of 110 patients.

As the primary tumor-aware ALPS sensitivity analysis, strict contralateral ALPS was used, restricted to the ALPS-valid survival cohort (N = 110). Tumor laterality was determined from segmentation masks; tumors were considered lateralized if at least 80% of voxels were located in one hemisphere. For left-sided tumors, right ALPS was analyzed; for right-sided tumors, left ALPS was used, since this restricts assessment to the hemisphere least likely to be affected by tumor-related diffusion changes. Six patients with bilateral or midline tumors (laterality <80%) were excluded; no additional cases were excluded for tumor-mask overlap with the contralateral ALPS region or its 5-mm safety margin, yielding a strict contralateral ALPS cohort of 104 patients. This two-tiered approach, bilateral tumor-aware exclusion followed by strict contralateral restriction, aimed to ensure that ALPS measurements reflected preserved perivascular diffusion rather than tumor-related contamination, and to test whether ALPS in the normal-appearing contralateral white matter provided a clearer prognostic signal in glioblastoma (20, 21).

### Machine-learning analysis for 12-month mortality prediction

In a secondary analysis, we investigated whether ASL radiomics or DTI-ALPS added extra predictive value for 12-month mortality classification beyond a volume-aware clinical baseline. The endpoint was death within 365 days after imaging: such death was labeled as positive, survival beyond 365 days as negative, and patients censored before 365 days were excluded. Model blocks were structured factorially: core clinical variables alone; core variables plus total tumor volume; core variables plus ASL radiomics; and core variables plus both tumor volume and ASL radiomics. This setup allowed assessment of the incremental value of ASL radiomics beyond the clinical baseline and volume-aware baseline. Similarly, DTI-ALPS incremental value was tested in the ALPS-valid cohort. The classifiers used included L2-regularized logistic regression, elastic-net logistic regression, linear support vector machine, RBF support vector machine, random forest, and extra-trees. Performance was measured by the patient-level AUC with 95% confidence intervals (via bootstrap with over 2,000 iterations), the change in AUC after adding imaging biomarkers, the Brier score with confidence intervals, and the precision-recall AUC to account for class imbalance. All pairwise model comparisons were adjusted using the Benjamini-Hochberg false-discovery-rate correction.

### Machine-learning explainability analysis

Permutation feature importance for the ASL-only random forest model was used to characterize which features contributed most to the model’s predictions, independent of whether that model demonstrated incremental value over the clinical baseline (see Results). In each cross-validation fold, preprocessing and feature selection were performed only on the training data. The model was then tested on the held-out fold to determine the baseline ROC AUC. Each selected ASL radiomic feature was permuted in the test fold, and the resulting decrease in ROC AUC was recorded. This process was repeated for all 5 × 5 cross-validation folds, and features were ranked by the average decrease in ROC AUC across folds.

### Survival analysis

**Figure 2:**
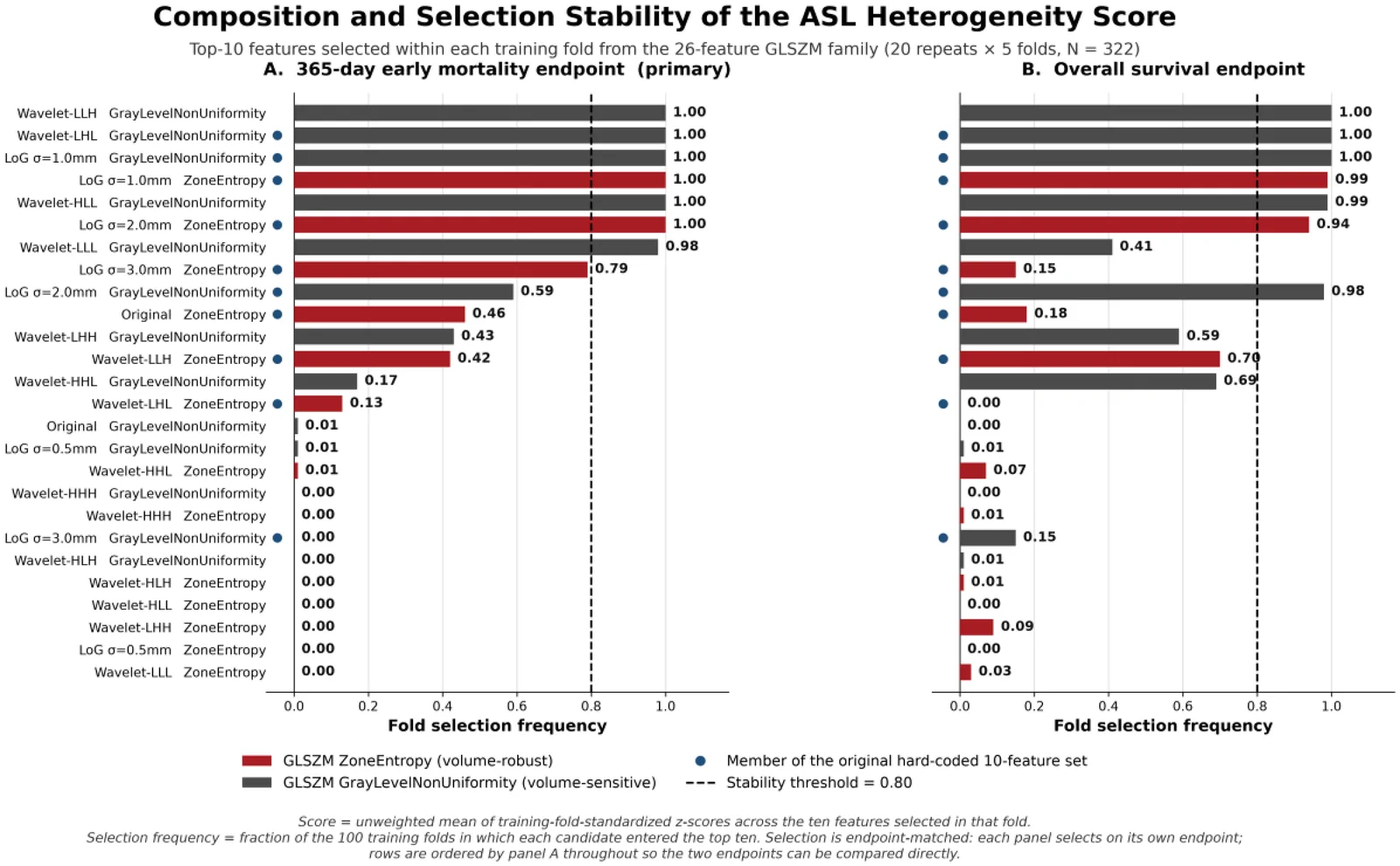
Composition and selection stability of the ASL heterogeneity score. The heterogeneity score comprises 10 fixed GLSZM features (6 ZoneEntropy and 4 GrayLevelNonUniformity variants across original, Laplacian-of-Gaussian–filtered, and wavelet-filtered ASL images), combined as an unweighted mean of training-fold-standardized z-scores. Bars show the frequency (0–1) with which each feature was independently re-selected as a top 10 feature when selection was repeated in a data-driven manner within each cross-validation fold, drawn from the full 26-feature GLSZM ZoneEntropy/GrayLevelNonUniformity family (N = 322). On the 365-day endpoint six features were selected in every fold, a seventh in 98% and an eighth in 79% (right of the dashed line), indicating high selection stability, while the remaining candidates were selected less consistently. Three features outside the ten emphasized by earlier classifier work were selected more often than four features within it, so the score’s prognostic signal is carried by a stable subset rather than by any fixed ten-feature list. Filled markers indicate membership of that earlier ten-feature set.

Cox proportional hazards models were used to analyze the association between overall survival and imaging predictors, including an ASL heterogeneity score, a low-ALPS score, and their interaction. The ASL heterogeneity score was derived within each cross-validation fold rather than fixed in advance. Candidates were drawn from a pre-specified 26-feature family comprising every GLSZM ZoneEntropy and GrayLevelNonUniformity variant across original, LoG-filtered, and wavelet-filtered ASL images. Every subsequent step was confined to training folds: within each of 100 training folds (20 repeats of 5-fold cross-validation) the ten candidates with the strongest training-fold univariate Cox association were selected, missing values were imputed using training-fold medians, and features were standardized using training-fold means and standard deviations. Each held-out patient’s score is the unweighted mean of their training-fold-standardized selected features; held-out scores were pooled across folds and averaged across repeats to give one out-of-fold score per patient. Selection was endpoint-matched, so the overall-survival and 365-day analyses each selected on their own endpoint. Because the selected set varies by fold, the score is not a fixed ten-feature list: on the 365-day endpoint six candidates entered in every fold, a seventh in 98% and an eighth in 79%, and three features outside the ten emphasized by earlier classifier work were selected more often than four features within it (**Figure 2**). To test whether the pre-specified family itself was carrying the result, the entire procedure was repeated with the candidate pool widened to all 208 GLSZM features, to all 234 Entropy and NonUniformity features of any texture family, and finally to all 1,209 non-shape ASL features screened inside each training fold, so that no aspect of the candidate pool was chosen on the full cohort. Although normalized size-zone features such as SizeZoneNonUniformityNormalized are inherently volume-robust by construction, they were not among the ten features constituting the final score, which instead relied on ZoneEntropy and GrayLevelNonUniformity variants, with volume-robustness assessed empirically rather than assumed from feature normalization alone. Higher scores indicated greater spatial heterogeneity of ASL perfusion within the tumor. Because several radiomic texture features can be numerically sensitive to tumor volume even after excluding explicit shape features, total tumor volume was quantified from the segmentation mask, natural-logarithm transformed, standardized, and included as a single log-volume term in all Cox models testing the ASL heterogeneity score. For clarity, every analysis described as volume-adjusted in this manuscript adjusts for log-transformed tumor volume, unless an alternative volume specification is stated explicitly.

Because the heterogeneity score was strongly correlated with tumor volume, four further analyses were specified after peer review to test whether the early-mortality association could reflect residual or non-linear volume confounding. First, tumor volume was modeled flexibly, using restricted cubic splines with three, four, and five knots (Harrell knot placement), quadratic and cubic polynomial terms, and non-parametric quintile and decile indicators, in place of the single log-volume term used in the primary model. Second, coefficient stability was assessed in Cox models stratified on volume tertiles, quartiles, and quintiles, within individual volume tertiles, and through a score-by-volume interaction term, with between-stratum heterogeneity quantified using Cochran’s Q and I². Third, the heterogeneity score was orthogonalized with respect to volume by regressing it on volume (linear, polynomial, restricted-cubic-spline, and rank-based specifications) and refitting the Cox model on the residual score, which is uncorrelated with volume by construction. Fourth, a negative-control calibration procedure quantified how often the volume-adjusted model would reject for a variable carrying the score’s volume relationship but no independent prognostic content: 2,000 synthetic scores were generated by Gaussian copula matched to the observed score-volume Spearman correlation, and 2,000 permutations of the observed score were generated within volume deciles, each refitted under both the log-volume and the five-knot-spline volume adjustment.

Separately, because patients censored before 365 days were older than those retained, the primary 365-day model was refitted with stabilized inverse-probability-of-censoring weights estimated from age, sex, MGMT promoter methylation status, extent of resection, and log tumor volume, with confidence intervals obtained by patient-level bootstrap (500 replicates). A complete-case analysis restricted to patients with known 12-month status was also performed.

The contralateral ALPS measurement was standardized to z-scores and reverse-coded to generate a low-ALPS phenotype score, so that higher scores represented lower original contralateral ALPS. This adjustment aligned ALPS with the direction of the ASL heterogeneity score: higher heterogeneity indicates a more adverse perfusion phenotype, while higher low-ALPS scores suggest a more adverse ALPS phenotype. Both scores were scaled so that higher values reflected the hypothesized higher-risk imaging state. Cox models adjusted for age at MRI, sex, MGMT promoter methylation status, extent of resection, and tumor volume.

Proportional-hazards assumptions were formally tested using Schoenfeld residuals for all Cox models. Because the ASL heterogeneity score violated the proportional-hazards assumption over the full follow-up period, its association with mortality was further characterized using a time-varying-coefficient analysis across three follow-up windows (0–180, 180–365, and >365 days), and the hazard ratio at 365 days is reported as a time-restricted estimate, consistent with the window over which proportional hazards held. Continuous predictors (age, ASL heterogeneity score) were also tested for non-linearity using a quadratic-term likelihood-ratio test against the linear specification.

For exploratory analysis, Kaplan-Meier estimates were used to assess survival differences by ASL heterogeneity and contralateral ALPS status. Patients were divided into four groups: high ASL heterogeneity with low contralateral ALPS; high ASL heterogeneity with high contralateral ALPS; low ASL heterogeneity with low contralateral ALPS; and low ASL heterogeneity with high contralateral ALPS.

### Statistical analysis

Continuous variables were summarized using means or medians, as appropriate. Classifier performance was evaluated across four model blocks (core clinical variables; core clinical plus tumor volume; core clinical plus ASL radiomics; core clinical plus tumor volume and ASL radiomics) and summarized using AUC with bootstrap-derived 95% confidence intervals (patient-level resampling, stratified by class, ≥2000 iterations), Brier score, and precision-recall AUC given class imbalance. Survival associations were summarized using hazard ratios and 95% confidence intervals, with the proportional hazards assumption tested as described above. Kaplan-Meier curves were used for descriptive visualization of ASL/contralateral-ALPS phenotypes. Benjamini-Hochberg false-discovery-rate correction was applied across all pairwise model-block comparisons and all classifier-cohort combinations, not a subset, and adjusted q-values are reported throughout.

### Code and reproducibility

In accordance with TRIPOD+AI reporting recommendations for open science in prediction model studies (22),analysis code, a pinned and validated computational environment, and derived feature-selection outputs supporting this study are publicly available at https://github.com/Rafail99/ASL-ALPS.

## Results

### ASL perfusion heterogeneity was associated with early mortality beyond tumor volume

Higher ASL perfusion heterogeneity was associated with mortality within 365 days after imaging, after adjustment for tumor volume and clinical variables. Using a leakage-free, out-of-fold procedure in which the heterogeneity score’s constituent features were selected only within training folds and evaluated only on held-out patients, the ASL heterogeneity score was associated with 365-day mortality after adjustment for age, sex, MGMT promoter methylation status, extent of resection, and log-transformed total tumor volume (hazard ratio 1.39 per standard deviation increase, 95% confidence interval 1.07–1.81, p=0.015; N = 322, 90 events within 365 days).

Because several radiomic texture features can numerically encode tumor volume even after excluding explicit shape features, we tested whether this association was explained by tumor size. We also tested whether the association depended on the pre-specified candidate family. Repeating the entire out-of-fold procedure with the pool widened to all 208 GLSZM features gave a volume-adjusted 365-day hazard ratio of 1.37 (95% CI 1.04–1.81, p=0.027); widening to all 234 Entropy and NonUniformity features gave 1.37 (95% CI 1.05–1.79, p=0.019); and screening all 1,209 non-shape ASL features inside each training fold, so that no part of the candidate pool was chosen on the full cohort, gave 1.33 (95% CI 1.05–1.69, p=0.018). The early-mortality association therefore does not depend on any outcome-informed restriction of the feature space (**Supplementary Table 11**). Volume adjustment changed the estimate by only 3.2% (unadjusted hazard ratio 1.43, 95% CI 1.20–1.71; volume-adjusted 1.39, 95% CI 1.07–1.81), indicating that the early-mortality association was not attributable to tumor volume.

Because the heterogeneity score correlated strongly with tumor volume (Spearman ρ = 0.84), we tested directly whether residual or non-linear volume confounding, which a single adjustment term may not absorb, could account for this association (**Supplementary Table 12**). Modeling tumor volume flexibly did not change the estimate: across restricted cubic splines with three, four, and five knots, quadratic and cubic polynomial terms, and non-parametric volume quintile and decile indicators, the volume-adjusted 365-day hazard ratio ranged from 1.40 to 1.49 (all p<0.02; **Supplementary Table 12a**). The association was stable across the volume range: hazard ratios from Cox models stratified on volume tertiles, quartiles, and quintiles were 1.43, 1.37, and 1.41 respectively (all p<0.02), between-stratum heterogeneity was absent (Cochran Q p=0.85, I²=0%), and the score-by-volume interaction was null (hazard ratio 0.99, 95% CI 0.80–1.23, p=0.93; **Supplementary Table 12b**). When the score was orthogonalized with respect to volume, so that the residualized score was uncorrelated with volume by construction, the association persisted (hazard ratio 1.25–1.27 across linear, polynomial, spline, and rank-based residualization, all p<0.03; **Supplementary Table 12c**).

In a negative-control calibration procedure, synthetic scores matched to the observed score-volume correlation and permutations of the observed score within volume deciles — both of which carry the score’s relationship to volume but no independent prognostic content — reached the observed hazard ratio in 0.8% to 5.2% of 2,000 replicates, with type-I error at or near the nominal level in every configuration (3.9–5.3%; **Supplementary Table 12d**). Under the permutation null, which preserves the observed score-volume relationship most closely, the empirical p values were 0.008 and 0.014 under log-volume and spline adjustment respectively. Residual volume confounding is therefore unlikely to account for the early-mortality association, although one of the four configurations was borderline (empirical p=0.052).

Because 63 patients were censored before 365 days and were older than those retained (**Supplementary Table 8**), the primary model was refitted using stabilized inverse-probability-of-censoring weights. The weighted estimate was essentially unchanged (hazard ratio 1.40, 95% bootstrap CI 1.14–1.78), as was a complete-case analysis restricted to patients with known 12-month status (hazard ratio 1.38, 95% CI 1.07–1.79, N = 259; **Supplementary Table 13**).

**Figure 3:**
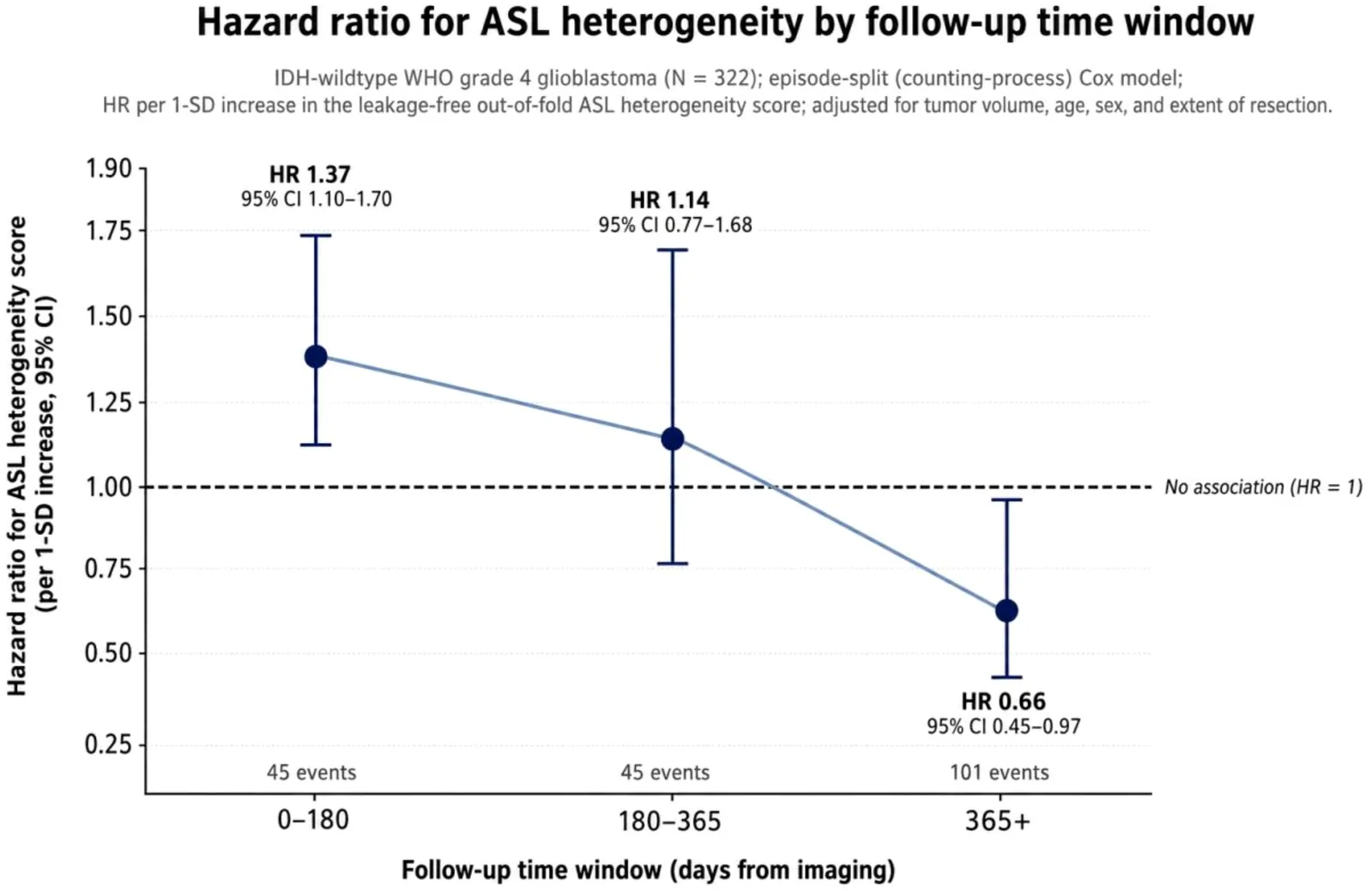
Hazard ratio for ASL heterogeneity by follow-up time window. Hazard ratios for the ASL heterogeneity score (per 1-SD increase) were estimated separately within three non-overlapping follow-up windows (0–180, 180–365, and >365 days from imaging) using window-split Cox proportional hazards models adjusted for tumor volume, age, sex, and extent of resection, in the primary survival cohort (N = 322). The hazard ratio was highest in the first 180 days (1.37, 95% CI 1.10–1.70) and attenuated progressively across follow-up (180–365 days: 1.14, 95% CI 0.77–1.68; >365 days: 0.66, 95% CI 0.45–0.97), consistent with a time-restricted, early-mortality-specific association rather than a constant effect across the full follow-up period. The estimate beyond one year falls below the null, which is most plausibly explained by depletion of susceptibles, patients with high heterogeneity surviving beyond a year being a selected subgroup, and should not be read as a protective effect. Event counts per window are shown below each estimate. Error bars represent 95% confidence intervals; the dashed line indicates no association (HR = 1).

Proportional-hazards testing showed that the hazard ratio for ASL heterogeneity was not constant across the full follow-up period (Schoenfeld p=0.007) but held within the 365-day window (Schoenfeld p=0.725; full covariate testing and non-linearity checks in **Supplementary Table 5**).A time-varying analysis across three follow-up windows showed a hazard ratio of approximately 1.4 within the first 180 days (95% CI 1.10–1.70), approximately 1.1 between 180 and 365 days (95% CI 0.77–1.68), and approximately 0.7 beyond one year (95% CI 0.45–0.97) (**Figure 3**). This pattern indicates that ASL perfusion heterogeneity carries a genuine, time-restricted prognostic signal concentrated in the early post-imaging period, rather than a constant effect across the full follow-up period.

### Overall survival, machine-learning, and phenotype-based associations were substantially attributable to tumor volume

Because several apparent prognostic and predictive associations in this study proved sensitive to tumor volume, we systematically tested each against volume adjustment. The unadjusted association between ASL heterogeneity and overall survival over the full follow-up period was no longer significant after adjustment for tumor volume (**Supplementary Table 3**). Machine-learning classification of 12-month mortality showed no incremental discriminative value from ASL radiomics beyond a clinical baseline that included tumor volume, across all six classifiers tested in either analytic cohort, after correction for multiple comparisons (**Supplementary Table 4**); tumor volume alone largely reproduced the apparent gain previously attributed to ASL radiomics. A four-group exploratory phenotype analysis combining ASL heterogeneity and contralateral ALPS status showed a graded survival pattern that did not remain significant after accounting for tumor volume (**Supplementary Figure 1**). High-heterogeneity group membership, a dichotomized construct, was correlated with tumor volume at Spearman ρ = 0.75–0.76; the continuous heterogeneity score correlated with volume at ρ = 0.84 (**Supplementary Table 12**). These findings indicate that tumor volume, rather than ASL radiomic texture per se, substantially explains these secondary associations and are reported in full in the Supplement.

### DTI-ALPS did not show independent prognostic or incremental predictive value

Contralateral DTI-ALPS was not independently associated with overall survival after adjustment for clinical covariates and ASL heterogeneity (hazard ratio 0.9–0.93 across sensitivity analyses, all p>0.5), and did not improve 12-month mortality classification beyond clinical variables or ASL radiomics in any classifier tested (**Supplementary Table 4**, S10). The heterogeneity-by-low-ALPS interaction term was also not significant in any analysis. Full classifier-level detail is reported in the Supplement.

## Discussion

In this study, ASL perfusion heterogeneity was associated with mortality within 365 days after imaging in patients with IDH-wildtype glioblastoma. Sensitivity analyses supported a modest component of this association that was not explained by tumor volume or clinical variables, rather than complete independence from tumor size. The association was time-restricted, strongest in the earliest months of follow-up and attenuating to null beyond one year. In contrast, the unadjusted association between ASL heterogeneity and overall survival across the full follow-up period and the apparent incremental value of ASL radiomics for 12-month mortality classification beyond clinical variables were both substantially attributable to tumor volume rather than to radiomic texture per se. Tumor-aware DTI-ALPS did not enhance classifier accuracy beyond clinical variables or ASL radiomics and was not independently associated with survival in the strict contralateral cohort. An exploratory four-group phenotype combining ASL heterogeneity and contralateral ALPS status showed a graded survival pattern that likewise did not remain significant after accounting for tumor volume.

The time-restricted association between ASL perfusion heterogeneity and early mortality is consistent with established biological understanding of glioblastoma. Glioblastoma exhibits varied perfusion characteristics across regions, reflecting abnormal angiogenesis, hypoxia, necrosis, infiltrative growth, and regional variation in vascular permeability and perfusion (23). ASL radiomic texture features quantify non-uniformity and entropy throughout the composite whole-tumor mask, which includes the surrounding FLAIR-abnormal region as well as enhancing and non-enhancing/necrotic tumor, and the permutation-importance analysis showed that the most influential ASL features were concentrated in heterogeneity-related texture measures, including GLSZM ZoneEntropy, GLSZM GrayLevelNonUniformity, GLDM low-gray-level emphasis features, and GLSZM ZoneVariance. However, several of these texture descriptors, including non-normalized gray-level non-uniformity measures, are numerically sensitive to tumor volume, and we found that the heterogeneity score correlated strongly with tumor volume (Spearman ρ=0.84). Notably, restricting the score to its most volume-robust constituent features (GLSZM ZoneEntropy alone, Spearman ρ with tumor volume = 0.71) did not decouple the signal from tumor size: the volume-adjusted early-mortality association was weaker and no longer significant using this restricted feature set (hazard ratio 1.24, 95% CI 0.89–1.73, p=0.211), compared with the full score (hazard ratio 1.39, p=0.015; full comparison in **Supplementary Table 6**).Two interpretations of this pattern are possible. The gray-level non-uniformity features excluded from the restricted set may carry prognostic information beyond tumor size; alternatively, and equally consistent with this comparison alone, they may retain residual volume signal that a single linear adjustment term does not fully absorb. Because the restricted-feature comparison cannot distinguish between these explanations, we tested them directly rather than inferring between them. Flexible volume modeling, stratification, orthogonalization, and negative-control calibration (**Supplementary Table 12**) each favored the first explanation, indicating that ASL perfusion heterogeneity carries a volume-independent component of prognostic information relevant to near-term risk, potentially reflecting a mixture of hyperperfused neovascular regions, hypoxic or necrotic low-perfusion areas, and disorganized tumor vascular architecture (24), while its association with longer-term survival is more difficult to disentangle from tumor size.

Our negative and limited independent DTI-ALPS findings should be interpreted in light of a deliberate methodological trade-off between statistical power and biological validity. DTI-ALPS indirectly reflects perivascular and neurofluid dynamics and is vulnerable to confounding by macrostructural tumor effects, including infiltration and edema (25). To mitigate this, we applied bilateral tumor-aware quality control, excluding cases in which the tumor extended into an ALPS region of interest or its safety margin, and further restricted the primary sensitivity analysis to the contralateral hemisphere. This reduced the cohort available for ALPS-specific analyses from 322 to 110 patients for survival analysis and 100 for machine learning; the primary ASL survival analysis retains all 322 patients, since it does not require an ALPS measurement. Patients with and without valid DTI-ALPS did not differ in age, sex, MGMT status, or extent of resection (**Supplementary Table 8**), indicating the ALPS-valid subgroup is representative of the primary cohort. The lack of independent significance of low contralateral ALPS in the Cox model and its limited classification value may be partly attributable to reduced power to detect a subtle secondary signal.

Our ASL findings build on previous research demonstrating that perfusion MRI holds prognostic value in glioblastoma. Earlier ASL studies indicated that tumor perfusion metrics correlate with progression-free and overall survival, supporting the use of non-contrast perfusion imaging for risk assessment (3). Radiomics research using perfusion MRI has further shown that quantitative imaging features can enhance survival predictions in glioblastoma (26). Our study extends this literature by explicitly testing, rather than assuming, that ASL-derived texture heterogeneity carries prognostic information not fully explained by tumor volume, using leakage-free feature selection and volume-adjusted modeling; we found this to be true specifically for early mortality risk, a more circumscribed finding than prior reports focused on general survival association. Regarding DTI-ALPS, the findings are more nuanced. Hagiwara et al. (2026) reported that contralateral ALPS and free-water DTI-derived metrics were associated with survival in IDH-wildtype glioblastoma. Similarly, Kim et al. reported the prognostic relevance of automated DTI-ALPS but noted that its performance was modest and that it lost significance once MGMT promoter methylation status was included in subset analyses (5). Our results align with this cautious interpretation: ALPS may reflect biologically meaningful neurofluid or white-matter microstructural information, but its independent prognostic value appears limited relative to established clinical and molecular predictors.

Our study offers several key strengths. First, to our knowledge, it is among the first to explicitly test whether radiomic prognostic signals in ASL perfusion imaging are attributable to tumor volume, rather than assuming shape-feature exclusion is sufficient to control for tumor size; this volume-adjustment approach may be broadly applicable to future radiomics studies. Second, the analysis focused solely on IDH-wildtype WHO grade 4 glioblastoma and patients who had gross-total or subtotal resection, reducing molecular and treatment-related variability. Third, the ASL heterogeneity score was constructed using a leakage-free, out-of-fold procedure in which feature selection, imputation, and standardization were all confined to training folds, avoiding the circularity risk of selecting features based on their association with the outcome subsequently tested. Critically, the association persisted when the candidate pool itself was chosen inside the folds, screening all 1,209 non-shape ASL features, so the finding does not depend on any outcome-informed restriction of the feature space. In addition, volume-independence was tested directly rather than assumed, through flexible volume modeling, stratification, orthogonalization, and a calibrated negative-control procedure in which synthetic and permuted scores carrying the same relationship to volume but no independent prognostic content did not reproduce the observed association. Fourth, proportional-hazards assumptions were formally tested rather than assumed, revealing a genuine time-varying structure that clarified the early-mortality specificity of the association between ASL heterogeneity and mortality. Finally, by transparently reporting the machine-learning classification and overall-survival findings as volume-confounded rather than omitting them, this study aims to provide a methodologically conservative and reproducible account of ASL radiomics’ prognostic value in this cohort.

Several limitations should be recognized. First, this study is retrospective, which introduces potential biases such as selection bias, missing data, and variability in imaging techniques and clinical management. Second, external validation has not been conducted; the partially volume-independent early-mortality association is preliminary until confirmed in independent, multi-center cohorts. We also did not directly compare the discriminative performance of our models against established clinical prognostic frameworks, such as the RTOG Recursive Partitioning Analysis classification. This comparison was not feasible in the present dataset because Karnofsky Performance Status, a required input for RPA classification, is not captured. More broadly, several clinically relevant variables were not available in this dataset, including Karnofsky Performance Status, steroid use, and adjuvant treatment completion. Molecular markers beyond MGMT were assessed but found non-informative in this IDH-wildtype grade-4 cohort and were not incorporated as covariates. Direct benchmarking against RPA or similar established staging systems is an important next step for evaluating whether ASL heterogeneity provides discriminative value beyond existing clinical frameworks, and should be prioritized in future prospective, multi-center studies where the necessary clinical variables, along with performance status and treatment-detail fields, are available. Third, radiomics features can be influenced by preprocessing steps, segmentation accuracy, scanner differences, and feature-selection methods; ASL volumes in this dataset were resampled to 1 mm isotropic resolution by the data provider prior to our analysis, and while a resolution-sensitivity analysis did not suggest the heterogeneity signal was an interpolation artifact, native-resolution data were not available to test this directly. Fourth, the ASL heterogeneity score was constructed from z-scored, normalized intensities and characterizes relative perfusion heterogeneity across the composite whole-tumor mask rather than absolute perfusion magnitude; a sensitivity analysis using non-normalized features did not preserve the volume-adjusted association, indicating the finding is specific to this normalized construct. Relatedly, because that mask combines enhancing tumor, non-enhancing/necrotic tumor, and surrounding FLAIR abnormality/edema, the score reflects perfusion heterogeneity across the entire segmented lesion, including the peritumoral FLAIR-abnormal region, and cannot be attributed specifically to the enhancing or non-enhancing tumor core; compartment-specific extraction was not performed and is an important direction for future work. Fifth, the association is modest in magnitude (hazard ratio approximately 1.4 per standard deviation) and the heterogeneity score remains strongly correlated with tumor volume (Spearman rho 0.84). Although the estimate proved robust to how the feature space is defined and to how volume is modeled, the volume-orthogonalized estimate (hazard ratio 1.25–1.27) is smaller than the primary estimate, indicating that part of the score’s prognostic signal is shared with tumor volume and that the independent contribution, while present, is modest. Sixth, age showed evidence of a non-linear association with survival in this larger cohort (quadratic-term likelihood-ratio test p=0.007); the primary model retains a linear age term for interpretability, and the heterogeneity score’s hazard ratio was materially unchanged when quadratic or cubic age terms were added. Seventh, DTI-ALPS is an indirect indicator and should not be regarded as a direct measure of glymphatic function; even strict contralateral ALPS may be affected by remote tumor effects, subclinical infiltration, age-related white matter changes, and diffusion scan parameters. Eighth, the machine-learning classification analysis, while methodologically rigorous, was underpowered to detect incremental value from any imaging-derived block beyond a volume-aware clinical baseline at this sample size, and this null result should not be interpreted as definitive evidence against any classification value for ASL radiomics. Finally, the four-group ASL-ALPS phenotype analysis was exploratory, based on dichotomized thresholds, did not remain significant after adjustment for volume, and should not be used for clinical risk stratification.

In summary, this study found that ASL perfusion radiomic heterogeneity was associated with early (365-day) mortality in IDH-wildtype glioblastoma, with a modest component of this association not attributable to tumor volume, and with a time-restricted effect that attenuated beyond one year. Associations with overall survival and incremental machine-learning classification value were substantially attributable to tumor volume rather than radiomic texture. Tumor-aware DTI-ALPS did not offer additional predictive or survival insight in this cohort. Given the retrospective, single-institution design and absence of external validation, these findings are hypothesis-generating. Future prospective, multicenter studies are needed to confirm the modest volume-independent component of the early-mortality prognostic value of ASL heterogeneity radiomics and to determine whether this signal generalizes across scanners and populations.

## Data Availability

Publicly available datasets were analyzed in this study. This data can be found here: Repository Name: The Cancer Imaging Archive (TCIA), Dataset Collection Name: The University of California San Francisco Preoperative Diffuse Glioma MRI Dataset (UCSF-PDGM), Direct Link: https://www.cancerimagingarchive.net/collection/ucsf-pdgm/, Digital Object Identifier (DOI): https://doi.org/10.7937/tcia.bdgf-8v37.
