# Supplementary material for: ASL perfusion radiomic heterogeneity is associated with early mortality in IDH-wildtype glioblastoma, beyond tumor volume: a time-varying effect with tumor-aware DTI-ALPS
Source: Front Oncol. 2026 Sep 9;16:1918963. doi: 10.3389/fonc.2026.1918963 (PMC13597259; doi:10.3389/fonc.2026.1918963)
Supplement: Supplementary file 1 [file DataSheet1.docx]

Supplementary Material

# Table S1. Software and Library Versions

| Package | Version |
| --- | --- |
| Python | 3.12.1 |
| PyRadiomics | 3.1.0 |
| lifelines | 0.30.3 |
| scikit-learn | 1.9.0 |
| SimpleITK | 2.5.4 |
| pandas | 2.3.3 |
| numpy | 1.26.4 |
| scipy | 1.17.1 |
| statsmodels | 0.14.6 |
| nibabel | 5.4.2 |
| matplotlib | 3.11.0 |

# Table S2. Radiomic Feature Extraction Parameters

| Parameter | Value |
| --- | --- |
| Total ASL features extracted | 1,223 |
| Shape-family features excluded | 14 |
| Non-shape features used in analysis | 1,209 |
| Gray-level discretization | Fixed bin width = 25 |
| Laplacian-of-Gaussian sigma values | 0.5, 1.0, 2.0, 3.0 mm |
| Wavelet decomposition | All 8 directional sub-bands |
| Image resampling | 1×1×1 mm isotropic |
| IBSI conformance | Not formally tested (PyRadiomics implements IBSI-standardized definitions) |

# Table S3. Overall-Survival Cox Model (Secondary/Sensitivity). ASL heterogeneity rows are fitted in the primary cohort (N=322); low-ALPS and interaction rows require a valid DTI-ALPS measurement and are fitted in the ALPS-valid subset (N=110).

| Analysis | N / Events | Predictor | HR | 95% CI | p |
| --- | --- | --- | --- | --- | --- |
| Overall survival, unadjusted | 322 / 191 | ASL heterogeneity score | 1.15 | 0.99–1.35 | 0.073 |
| Overall survival, volume-adjusted | 322 / 191 | ASL heterogeneity score | 1.17 | 0.92–1.49 | 0.203 |
| Overall survival (ALPS-valid subset) | 110 / 79 | Low contralateral ALPS score | 0.95 | 0.74–1.21 | 0.660 |
| Overall survival (ALPS-valid subset) | 110 / 79 | Heterogeneity × low-ALPS interaction | 0.97 | 0.78–1.20 | 0.765 |

*Note. The overall-survival association between ASL heterogeneity and mortality did not remain significant after adjustment for total tumor volume, indicating this association is substantially attributable to tumor size rather than radiomic texture. This contrasts with the 365-day early-mortality association reported as the primary finding (main text), which survived volume adjustment.*

# Table S4. Full 12-Month Mortality Classification Results (4-Block, Unified FDR)

## S4a. Performance Metrics — Cohort N=259 (Primary)

| Classifier | Block | AUC (95% CI) | PR-AUC (95% CI) | Brier (95% CI) |
| --- | --- | --- | --- | --- |
| logistic_l2 | core | 0.706 (0.639–0.771) | 0.557 (0.481–0.651) | 0.219 (0.199–0.239) |
| logistic_l2 | core+vol | 0.726 (0.659–0.790) | 0.584 (0.507–0.680) | 0.213 (0.191–0.235) |
| logistic_l2 | core+ASL | 0.719 (0.653–0.783) | 0.601 (0.519–0.690) | 0.211 (0.188–0.236) |
| logistic_l2 | core+vol+ASL | 0.713 (0.647–0.778) | 0.597 (0.519–0.685) | 0.213 (0.189–0.238) |
| elastic_net | core | 0.706 (0.638–0.772) | 0.558 (0.478–0.652) | 0.219 (0.200–0.239) |
| elastic_net | core+vol | 0.727 (0.662–0.789) | 0.584 (0.500–0.673) | 0.213 (0.193–0.234) |
| elastic_net | core+ASL | 0.721 (0.656–0.782) | 0.600 (0.517–0.688) | 0.210 (0.186–0.233) |
| elastic_net | core+vol+ASL | 0.717 (0.652–0.784) | 0.598 (0.520–0.682) | 0.211 (0.188–0.234) |
| linear_svm | core | 0.705 (0.639–0.766) | 0.558 (0.477–0.646) | 0.203 (0.189–0.216) |
| linear_svm | core+vol | 0.726 (0.659–0.785) | 0.584 (0.502–0.680) | 0.198 (0.183–0.213) |
| linear_svm | core+ASL | 0.716 (0.646–0.779) | 0.600 (0.522–0.684) | 0.198 (0.183–0.212) |
| linear_svm | core+vol+ASL | 0.711 (0.646–0.774) | 0.593 (0.519–0.679) | 0.199 (0.185–0.212) |
| rbf_svm | core | 0.718 (0.652–0.781) | 0.579 (0.502–0.675) | 0.201 (0.188–0.215) |
| rbf_svm | core+vol | 0.724 (0.663–0.786) | 0.543 (0.470–0.638) | 0.200 (0.187–0.214) |
| rbf_svm | core+ASL | 0.732 (0.670–0.793) | 0.565 (0.489–0.667) | 0.197 (0.185–0.210) |
| rbf_svm | core+vol+ASL | 0.727 (0.660–0.789) | 0.565 (0.487–0.661) | 0.198 (0.186–0.212) |
| random_forest | core | 0.665 (0.594–0.734) | 0.513 (0.444–0.617) | 0.223 (0.201–0.248) |
| random_forest | core+vol | 0.733 (0.670–0.791) | 0.558 (0.482–0.655) | 0.197 (0.180–0.215) |
| random_forest | core+ASL | 0.736 (0.667–0.798) | 0.574 (0.494–0.678) | 0.194 (0.175–0.213) |
| random_forest | core+vol+ASL | 0.731 (0.665–0.790) | 0.564 (0.488–0.669) | 0.195 (0.178–0.214) |
| extra_trees | core | 0.687 (0.619–0.754) | 0.531 (0.454–0.635) | 0.219 (0.201–0.237) |
| extra_trees | core+vol | 0.731 (0.669–0.790) | 0.545 (0.473–0.651) | 0.210 (0.196–0.223) |
| extra_trees | core+ASL | 0.730 (0.659–0.794) | 0.565 (0.491–0.670) | 0.202 (0.184–0.219) |
| extra_trees | core+vol+ASL | 0.728 (0.664–0.791) | 0.562 (0.489–0.661) | 0.202 (0.186–0.220) |

*Event rate: 0.347 (90/259)*

## S4a (cont.). Performance Metrics — Cohort N=100 (ALPS-Valid)

| Classifier | Block | AUC (95% CI) | PR-AUC (95% CI) | Brier (95% CI) |
| --- | --- | --- | --- | --- |
| logistic_l2 | core | 0.762 (0.663–0.854) | 0.617 (0.504–0.773) | 0.200 (0.165–0.239) |
| logistic_l2 | core+vol | 0.781 (0.675–0.875) | 0.673 (0.549–0.817) | 0.188 (0.148–0.230) |
| logistic_l2 | core+ASL | 0.797 (0.694–0.884) | 0.757 (0.654–0.857) | 0.182 (0.140–0.226) |
| logistic_l2 | core+vol+ASL | 0.787 (0.691–0.873) | 0.743 (0.636–0.843) | 0.187 (0.145–0.230) |
| elastic_net | core | 0.757 (0.653–0.851) | 0.607 (0.493–0.762) | 0.200 (0.167–0.236) |
| elastic_net | core+vol | 0.785 (0.686–0.876) | 0.676 (0.552–0.822) | 0.188 (0.152–0.225) |
| elastic_net | core+ASL | 0.800 (0.694–0.890) | 0.749 (0.637–0.849) | 0.178 (0.141–0.220) |
| elastic_net | core+vol+ASL | 0.794 (0.694–0.884) | 0.747 (0.642–0.848) | 0.180 (0.141–0.220) |
| linear_svm | core | 0.764 (0.662–0.855) | 0.620 (0.508–0.773) | 0.193 (0.173–0.215) |
| linear_svm | core+vol | 0.782 (0.680–0.873) | 0.677 (0.549–0.818) | 0.189 (0.169–0.210) |
| linear_svm | core+ASL | 0.788 (0.683–0.875) | 0.742 (0.621–0.850) | 0.177 (0.149–0.207) |
| linear_svm | core+vol+ASL | 0.773 (0.668–0.870) | 0.723 (0.604–0.837) | 0.182 (0.156–0.209) |
| rbf_svm | core | 0.774 (0.668–0.869) | 0.663 (0.539–0.808) | 0.190 (0.168–0.214) |
| rbf_svm | core+vol | 0.769 (0.672–0.859) | 0.655 (0.537–0.796) | 0.197 (0.180–0.212) |
| rbf_svm | core+ASL | 0.786 (0.681–0.871) | 0.744 (0.633–0.844) | 0.175 (0.147–0.202) |
| rbf_svm | core+vol+ASL | 0.769 (0.663–0.863) | 0.737 (0.617–0.841) | 0.181 (0.155–0.207) |
| random_forest | core | 0.746 (0.635–0.840) | 0.606 (0.481–0.747) | 0.197 (0.163–0.236) |
| random_forest | core+vol | 0.795 (0.698–0.881) | 0.699 (0.578–0.824) | 0.179 (0.153–0.206) |
| random_forest | core+ASL | 0.748 (0.649–0.842) | 0.678 (0.564–0.807) | 0.187 (0.153–0.221) |
| random_forest | core+vol+ASL | 0.740 (0.633–0.838) | 0.647 (0.516–0.788) | 0.191 (0.156–0.227) |
| extra_trees | core | 0.782 (0.686–0.870) | 0.701 (0.593–0.818) | 0.191 (0.166–0.217) |
| extra_trees | core+vol | 0.808 (0.710–0.886) | 0.726 (0.612–0.837) | 0.196 (0.180–0.215) |
| extra_trees | core+ASL | 0.751 (0.638–0.849) | 0.681 (0.561–0.815) | 0.188 (0.159–0.220) |
| extra_trees | core+vol+ASL | 0.748 (0.638–0.851) | 0.672 (0.548–0.811) | 0.190 (0.162–0.219) |

*Event rate: 0.36 (36/100)*

## S4b. Block Comparisons — Unified BH-FDR Across All 48 Comparisons (N=259)

| Classifier | Comparison | ΔAUC | 95% CI | p | q |
| --- | --- | --- | --- | --- | --- |
| logistic_l2 | core+vol vs core | 0.020 | [−0.017, 0.054] | 0.274 | 0.834 |
| logistic_l2 | core+ASL vs core | 0.013 | [−0.032, 0.062] | 0.528 | 0.890 |
| logistic_l2 | core+vol+ASL vs core+vol | −0.013 | [−0.052, 0.021] | 0.505 | 0.890 |
| logistic_l2 | core+vol+ASL vs core+ASL | −0.007 | [−0.016, 0.002] | 0.152 | 0.834 |
| elastic_net | core+vol vs core | 0.021 | [−0.015, 0.056] | 0.259 | 0.834 |
| elastic_net | core+ASL vs core | 0.015 | [−0.032, 0.058] | 0.569 | 0.890 |
| elastic_net | core+vol+ASL vs core+vol | −0.010 | [−0.042, 0.022] | 0.591 | 0.890 |
| elastic_net | core+vol+ASL vs core+ASL | −0.004 | [−0.011, 0.003] | 0.331 | 0.834 |
| linear_svm | core+vol vs core | 0.021 | [−0.016, 0.057] | 0.260 | 0.834 |
| linear_svm | core+ASL vs core | 0.011 | [−0.035, 0.058] | 0.625 | 0.901 |
| linear_svm | core+vol+ASL vs core+vol | −0.015 | [−0.057, 0.022] | 0.417 | 0.834 |
| linear_svm | core+vol+ASL vs core+ASL | −0.005 | [−0.013, 0.003] | 0.265 | 0.834 |
| rbf_svm | core+vol vs core | 0.006 | [−0.033, 0.044] | 0.777 | 0.956 |
| rbf_svm | core+ASL vs core | 0.014 | [−0.048, 0.072] | 0.667 | 0.915 |
| rbf_svm | core+vol+ASL vs core+vol | 0.003 | [−0.048, 0.051] | 0.879 | 0.974 |
| rbf_svm | core+vol+ASL vs core+ASL | −0.005 | [−0.015, 0.005] | 0.309 | 0.834 |
| random_forest | core+vol vs core | 0.069 | [0.018, 0.119] | 0.009 | 0.432 |
| random_forest | core+ASL vs core | 0.071 | [−0.006, 0.147] | 0.066 | 0.834 |
| random_forest | core+vol+ASL vs core+vol | −0.003 | [−0.060, 0.057] | 0.911 | 0.974 |
| random_forest | core+vol+ASL vs core+ASL | −0.005 | [−0.018, 0.007] | 0.382 | 0.834 |
| extra_trees | core+vol vs core | 0.044 | [0.007, 0.084] | 0.022 | 0.528 |
| extra_trees | core+ASL vs core | 0.043 | [−0.030, 0.117] | 0.243 | 0.834 |
| extra_trees | core+vol+ASL vs core+vol | −0.003 | [−0.053, 0.051] | 0.949 | 0.990 |
| extra_trees | core+vol+ASL vs core+ASL | −0.002 | [−0.011, 0.007] | 0.708 | 0.944 |

## S4b (cont.). Block Comparisons — Unified BH-FDR (N=100)

| Classifier | Comparison | ΔAUC | 95% CI | p | q |
| --- | --- | --- | --- | --- | --- |
| logistic_l2 | core+vol vs core | 0.019 | [−0.037, 0.079] | 0.513 | 0.890 |
| logistic_l2 | core+ASL vs core | 0.035 | [−0.047, 0.120] | 0.406 | 0.834 |
| logistic_l2 | core+vol+ASL vs core+vol | 0.006 | [−0.057, 0.065] | 0.840 | 0.965 |
| logistic_l2 | core+vol+ASL vs core+ASL | −0.010 | [−0.027, 0.006] | 0.233 | 0.834 |
| elastic_net | core+vol vs core | 0.028 | [−0.030, 0.086] | 0.375 | 0.834 |
| elastic_net | core+ASL vs core | 0.043 | [−0.042, 0.131] | 0.335 | 0.834 |
| elastic_net | core+vol+ASL vs core+vol | 0.009 | [−0.048, 0.065] | 0.771 | 0.956 |
| elastic_net | core+vol+ASL vs core+ASL | −0.006 | [−0.017, 0.003] | 0.289 | 0.834 |
| linear_svm | core+vol vs core | 0.017 | [−0.039, 0.076] | 0.593 | 0.890 |
| linear_svm | core+ASL vs core | 0.023 | [−0.061, 0.109] | 0.543 | 0.890 |
| linear_svm | core+vol+ASL vs core+vol | −0.009 | [−0.071, 0.053] | 0.797 | 0.956 |
| linear_svm | core+vol+ASL vs core+ASL | −0.015 | [−0.038, 0.007] | 0.174 | 0.834 |
| rbf_svm | core+vol vs core | −0.005 | [−0.067, 0.059] | 0.913 | 0.974 |
| rbf_svm | core+ASL vs core | 0.012 | [−0.091, 0.110] | 0.844 | 0.965 |
| rbf_svm | core+vol+ASL vs core+vol | 0.000 | [−0.074, 0.075] | 0.994 | 0.994 |
| rbf_svm | core+vol+ASL vs core+ASL | −0.017 | [−0.044, 0.009] | 0.202 | 0.834 |
| random_forest | core+vol vs core | 0.049 | [−0.034, 0.134] | 0.263 | 0.834 |
| random_forest | core+ASL vs core | 0.003 | [−0.119, 0.131] | 0.992 | 0.994 |
| random_forest | core+vol+ASL vs core+vol | −0.055 | [−0.135, 0.024] | 0.175 | 0.834 |
| random_forest | core+vol+ASL vs core+ASL | −0.008 | [−0.031, 0.015] | 0.473 | 0.890 |
| extra_trees | core+vol vs core | 0.026 | [−0.034, 0.090] | 0.412 | 0.834 |
| extra_trees | core+ASL vs core | −0.030 | [−0.153, 0.094] | 0.638 | 0.901 |
| extra_trees | core+vol+ASL vs core+vol | −0.059 | [−0.146, 0.030] | 0.192 | 0.834 |
| extra_trees | core+vol+ASL vs core+ASL | −0.003 | [−0.020, 0.013] | 0.740 | 0.956 |

*Note. Benjamini-Hochberg false-discovery-rate correction was applied jointly across all 48 pairwise block comparisons spanning both cohorts. No comparison reached significance (minimum q=0.432, random forest core+volume vs. core, N=259). The comparison most directly testing incremental ASL value beyond tumor volume (core+volume+ASL vs. core+volume) was near zero or negative across all twelve classifier-cohort combinations, confirming that ASL radiomics does not add discriminative value beyond a volume-aware clinical baseline in this cohort. AUC and PR-AUC for linear and RBF SVM classifiers are computed from decision-function scores; Brier scores for these classifiers required probability calibration (Platt/sigmoid scaling, fit within training folds only) and may reflect a marginally different score distribution than the decision-function-based discrimination metrics.*

# Table S5. Proportional-Hazards and Non-Linearity Testing (primary model N=322; interaction model N=110)

| Covariate | Primary volume-adjusted model, N=322 (overall follow-up) | Primary volume-adjusted model, N=322 (365-day window) |
| --- | --- | --- |
| ASL heterogeneity score | 0.007 (violated) | 0.725 (holds) |
| Age at MRI | 0.040 | 0.270 |
| Sex | 0.551 | 0.380 |
| MGMT positive | 0.232 | 0.551 |
| MGMT unknown/indeterminate | 0.045 | 0.280 |
| Extent of resection (GTR) | 0.194 | 0.142 |
| Log tumor volume | 0.445 | 0.279 |
| Global test | χ²=19.53, df=7, p=0.007 | χ²=6.96, df=7, p=0.433 |
| Heterogeneity × low-ALPS interaction (ALPS-valid subset, N=110) | 0.088 | 0.436 |
| Global (interaction model; ALPS-valid subset, N=110) | p=0.001 | p=0.390 |

## Non-Linearity (Quadratic-Term Likelihood-Ratio Test vs. Linear Specification, N=110)

| Predictor | χ² | p | Conclusion |
| --- | --- | --- | --- |
| Age | 7.196 | 0.007 | Evidence of non-linearity |
| ASL heterogeneity score | 1.835 | 0.175 | Linear assumption reasonable |

*Note. Proportional hazards were violated for the ASL heterogeneity score over the full follow-up period but held within the 365-day window (p=0.725), motivating the time-varying characterization presented in the main text (Figure 3). Extent of resection no longer violates the proportional-hazards assumption within the 365-day window in the larger cohort (p=0.142). Age showed evidence of a non-linear association with survival (quadratic-term likelihood-ratio test χ²=7.196, p=0.007); the primary model retains a linear age term for interpretability, and the heterogeneity score's hazard ratio was materially unchanged when quadratic or cubic age terms were added. The heterogeneity score itself showed no evidence of non-linearity (p=0.175).*

# Table S6. Volume-Robust vs. Full Heterogeneity Score Comparison (N=322)

| Score | Analysis | In-Sample HR | OOF HR | Volume-Adjusted HR | Score–Volume ρ |
| --- | --- | --- | --- | --- | --- |
| Full (top-10 of 26) | Overall | 1.19 (1.02–1.39), p=0.029 | 1.15 (0.99–1.35), p=0.073 | 1.17 (0.92–1.49), p=0.203 | 0.849 |
| Full (top-10 of 26) | 365-day | 1.47 (1.23–1.76), p=0.001 | 1.43 (1.20–1.71), p&lt;0.001 | 1.39 (1.07–1.81), p=0.015 | 0.843 |
| Volume-robust (top-6 ZoneEntropy) | Overall | 1.06 (0.91–1.25), p=0.461 | 1.04 (0.89–1.23), p=0.588 | 0.95 (0.76–1.19), p=0.630 | 0.707 |
| Volume-robust (top-6 ZoneEntropy) | 365-day | 1.42 (1.12–1.80), p=0.004 | 1.42 (1.12–1.80), p=0.004 | 1.24 (0.89–1.73), p=0.211 | 0.709 |

*Note. Restricting the heterogeneity score to its most volume-robust constituent features (GLSZM ZoneEntropy alone) did not decouple the signal from tumor volume and weakened the volume-adjusted early-mortality association below statistical significance, indicating that the excluded GrayLevelNonUniformity features contribute to the signal. This comparison alone cannot distinguish prognostic information beyond tumor size from residual volume signal; the two explanations are separated directly in Supplementary Table S12. The volume-independent early-mortality signal is therefore a property of the full ten-feature score rather than any single volume-robust feature subset.*

# Table S7. Resolution- and Normalization-Sensitivity Analyses (ALPS-valid subset, N=110)

| Analysis | Score–Standard Correlation | In-Sample 365-Day HR | OOF 365-Day HR | Volume-Adjusted 365-Day HR |
| --- | --- | --- | --- | --- |
| Standard pipeline (1mm, z-scored) | — (reference) | 2.09 (1.49–2.91), p<0.001 | 1.96 (1.39–2.77), p<0.001 | 2.22 (1.26–3.91), p=0.005 |
| Coarse resolution (~3mm surrogate) | r=0.94 | 1.94 (1.40–2.67), p<0.001 | 1.90 (1.38–2.63), p<0.001 | 2.68 (1.39–5.17), p=0.003 |
| Non-normalized (absolute CBF) | r=0.76 | 1.59 (1.18–2.15), p=0.003 | 1.49 (1.10–2.01), p=0.010 | 1.14 (0.66–1.99), p=0.636 |

*Note. The resolution-sensitivity analysis, coarsening ASL images to approximately 3 mm (typical of native pseudo-continuous ASL acquisition), preserved the volume-adjusted early-mortality association, indicating the primary finding is not attributable to interpolation-manufactured texture. Removing intensity normalization (using absolute, non-z-scored cerebral blood flow values instead of within-mask z-scores) abolished the volume-adjusted association, indicating the score's prognostic value is specific to relative, normalized perfusion heterogeneity rather than absolute perfusion magnitude. Native-resolution ASL data were not available in the UCSF-PDGM release to test resolution-sensitivity directly; the coarse-resolution analysis serves as a surrogate. These preprocessing sensitivity analyses were performed in the ALPS-valid subset (N=110), the cohort for which coarse-resampled and non-normalized features were extracted, and the hazard ratios in this table are therefore not directly comparable with the N=322 primary estimates reported elsewhere.*

# Table S8. Baseline Characteristics, Included vs. Excluded Patients

## 363→322 (GTR/STR Exclusion)

| Variable | Included (n=322) | Excluded (n=41) | p |
| --- | --- | --- | --- |
| Age, median [IQR] | 62 [54–69] | 68 [55–75] | 0.063 (Mann-Whitney); 0.209 (Welch t) |
| Sex, n (% female) | 130 (40%) | 17 (41%) | 1.000 (Fisher) |
| MGMT, neg/pos/unknown (n) | 89/223/10 | 12/24/5 | 0.019 (χ²) |
| Extent of resection, GTR/STR/other (n) | 214/108/0 | 0/0/41 | By definition |

## 322→259 (Censored-Before-365-Days Exclusion)

| Variable | Included (n=259) | Excluded (n=63) | p |
| --- | --- | --- | --- |
| Age, median [IQR] | 61 [54–69] | 64 [59–72] | 0.032 (Mann-Whitney); 0.060 (Welch t) |
| Sex, n (% female) | 104 (40%) | 26 (41%) | 0.887 (Fisher) |
| MGMT, neg/pos/unknown (n) | 71/180/8 | 18/43/2 | 0.982 (χ²) |
| Extent of resection, GTR/STR (n) | 178/81 | 36/27 | 0.101 (Fisher) |

*Note. Extent of resection defines the 363→322 exclusion step (included = gross-total/subtotal resection; excluded = biopsy-only); group membership and the variable are identical, so no statistical comparison is applicable. KPS and tumor laterality fields are not available in the UCSF-PDGM dataset and could not be reported. 1p/19q codeletion status was not tabulated as a comparison, as all patients in this IDH-wildtype grade 4 cohort had 0% codeletion (non-informative). MGMT status distribution differed significantly between included and excluded groups at the GTR/STR exclusion step (p=0.019). Patients excluded due to censoring before 365 days were significantly older than those retained (median 64 vs. 61 years, p=0.032), consistent with the selection-bias pattern described in Methods. This selection was shown to be inconsequential in an inverse-probability-of-censoring-weighted sensitivity analysis (Supplementary Table S13).*

322→110 (DTI-ALPS Quality-Control Exclusion)

| Variable | Included (n=110) | Excluded (n=212) | p |
| --- | --- | --- | --- |
| Age, median [IQR] | 62 [55–69] | 62 [54–69] | 0.431 (Mann-Whitney); 0.289 (Welch t) |
| Sex, n (% female) | 47 (43%) | 83 (39%) | 0.551 (Fisher) |
| MGMT, neg/pos/unknown (n) | 34/75/1 | 55/148/9 | 0.195 (χ²) |
| Extent of resection, GTR/STR (n) | 78/32 | 136/76 | 0.263 (Fisher) |

*Note. Patients whose DTI-ALPS processing passed bilateral tumour-aware quality control did not differ from those excluded on any measured baseline variable. The ALPS-valid subset used for the secondary DTI-ALPS analyses is therefore representative of the N=322 primary ASL survival cohort.*

# Table S9. Per-Fold Selected ASL Feature Frequencies (Machine-Learning Classification, N=259, 25 Folds)

| Selections (of 25) | Fraction | Feature |
| --- | --- | --- |
| 25 | 1.00 | asl__log-sigma-1-0-mm-3D_glszm_ZoneEntropy |
| 25 | 1.00 | asl__log-sigma-1-0-mm-3D_glszm_GrayLevelNonUniformity |
| 25 | 1.00 | asl__wavelet-LHL_glszm_GrayLevelNonUniformity |
| 24 | 0.96 | asl__log-sigma-3-0-mm-3D_glszm_ZoneEntropy |
| 18 | 0.72 | asl__wavelet-LHL_glrlm_RunLengthNonUniformity |
| 17 | 0.68 | asl__wavelet-LLH_glszm_ZoneEntropy |
| 15 | 0.60 | asl__wavelet-LLL_gldm_DependenceNonUniformity |
| 15 | 0.60 | asl__log-sigma-2-0-mm-3D_glszm_GrayLevelNonUniformity |
| 14 | 0.56 | asl__wavelet-HHL_glszm_ZoneVariance |
| 12 | 0.48 | asl__wavelet-LHL_glszm_ZoneEntropy |
| 12 | 0.48 | asl__wavelet-LLL_firstorder_Energy |
| 10 | 0.40 | asl__wavelet-LHH_glszm_GrayLevelNonUniformity |
| 8 | 0.32 | asl__wavelet-HHH_glszm_ZoneVariance |
| 6 | 0.24 | asl__log-sigma-2-0-mm-3D_glrlm_GrayLevelNonUniformity |
| 6 | 0.24 | asl__original_gldm_DependenceNonUniformity |
| 5 | 0.20 | asl__wavelet-LHL_glcm_Imc1 |
| 5 | 0.20 | asl__wavelet-HHL_glszm_GrayLevelNonUniformity |
| 5 | 0.20 | asl__wavelet-HLH_glszm_ZoneVariance |
| 4 | 0.16 | asl__original_gldm_LargeDependenceHighGrayLevelEmphasis |
| 4 | 0.16 | asl__log-sigma-3-0-mm-3D_glrlm_RunLengthNonUniformity |
| 4 | 0.16 | asl__wavelet-LHH_glrlm_RunLengthNonUniformity |
| 3 | 0.12 | asl__wavelet-HLH_glrlm_RunLengthNonUniformity |
| 3 | 0.12 | asl__original_glrlm_GrayLevelNonUniformity |
| 3 | 0.12 | asl__wavelet-LLL_glszm_GrayLevelNonUniformity |
| 3 | 0.12 | asl__log-sigma-1-0-mm-3D_glszm_SizeZoneNonUniformityNormalized |
| 2 | 0.08 | asl__wavelet-HHL_glszm_LargeAreaEmphasis |
| 2 | 0.08 | asl__log-sigma-3-0-mm-3D_glszm_GrayLevelNonUniformity |
| 2 | 0.08 | asl__wavelet-LLL_ngtdm_Coarseness |
| 2 | 0.08 | asl__original_glrlm_RunLengthNonUniformity |
| 2 | 0.08 | asl__wavelet-LLH_glszm_GrayLevelNonUniformity |
| 2 | 0.08 | asl__wavelet-LLL_glrlm_LongRunLowGrayLevelEmphasis |
| 1 | 0.04 | asl__wavelet-LLL_gldm_LowGrayLevelEmphasis |
| 1 | 0.04 | asl__original_firstorder_Energy |
| 1 | 0.04 | asl__wavelet-LLL_glrlm_LowGrayLevelRunEmphasis |
| 1 | 0.04 | asl__original_glszm_GrayLevelNonUniformity |
| 1 | 0.04 | asl__wavelet-HHL_glszm_SmallAreaEmphasis |
| 1 | 0.04 | asl__wavelet-LLL_glrlm_RunLengthNonUniformity |
| 1 | 0.04 | asl__wavelet-LLL_gldm_LargeDependenceLowGrayLevelEmphasis |
| 1 | 0.04 | asl__log-sigma-2-0-mm-3D_glrlm_RunLengthNonUniformity |
| 1 | 0.04 | asl__wavelet-LLL_firstorder_Kurtosis |
| 1 | 0.04 | asl__wavelet-LHL_glrlm_GrayLevelNonUniformity |
| 1 | 0.04 | asl__log-sigma-0-5-mm-3D_glszm_ZoneVariance |
| 1 | 0.04 | asl__wavelet-HHH_glcm_ClusterShade |
| 1 | 0.04 | asl__original_glszm_ZoneEntropy |
| 1 | 0.04 | asl__wavelet-HLL_glszm_ZoneEntropy |
| 1 | 0.04 | asl__log-sigma-0-5-mm-3D_glrlm_RunLengthNonUniformity |
| 1 | 0.04 | asl__original_gldm_LowGrayLevelEmphasis |
| 1 | 0.04 | asl__wavelet-HHH_glszm_SizeZoneNonUniformityNormalized |

*Note. Features are drawn from the full 1,209-feature non-shape ASL radiomic pool (all feature families and filter types), distinct from the fixed 10-feature GLSZM ZoneEntropy/GrayLevelNonUniformity set used to construct the primary heterogeneity score (Methods; Figure 2). SizeZoneNonUniformityNormalized, a volume-robust normalized feature, was selected in a minority of folds (≤0.12) but was not among the features used in the final heterogeneity score.*

### Table S10. ALPS Contribution in Machine-Learning Models (finalized, N=100)

| Model Type | Model Block | Finding | Interpretation |
| --- | --- | --- | --- |
| Linear | Core + ALPS logistic (L2) | Median coefficient +0.184 (positive in 84% of folds) | Small positive direction |
| Linear | Core + ALPS elastic-net logistic | Median coefficient +0.078 (positive in 80% of folds) | Small positive direction |
| Linear | Core + ALPS linear SVM | Median weight +0.094 (positive in 88% of folds) | Small positive direction |
| Linear | Core + ASL + ALPS logistic (L2) | Median coefficient +0.215 (positive in 88% of folds) | Similar direction after adding ASL |
| Linear | Core + ASL + ALPS elastic-net logistic | Median coefficient +0.075 (positive in 68% of folds) | Similar direction after adding ASL, less consistent |
| Linear | Core + ASL + ALPS linear SVM | Median weight +0.110 (positive in 92% of folds) | Similar direction after adding ASL |
| Tree-based | Core + ALPS random forest | Permutation AUC-drop median −0.0077; rank 2 of ~13 | Low importance; permuting ALPS slightly improved AUC |
| Tree-based | Core + ALPS extra-trees | Permutation AUC-drop median −0.0083; rank 4 of ~13 | Low importance; permuting ALPS slightly improved AUC |
| Tree-based | Core + ASL + ALPS random forest | Permutation AUC-drop median −0.0013; rank 10 of 14 | Negligible importance once ASL features present |
| Tree-based | Core + ASL + ALPS extra-trees | Permutation AUC-drop median −0.0033; rank 13 of 14 | Negligible importance once ASL features present |

**Note.** Across 25 cross-validation folds, the DTI-ALPS index made a negligible and unstable contribution to 12-month mortality prediction. In the linear models, its standardized coefficient was small in magnitude and positive in direction in the majority of folds under the final FSL-based pipeline (N=100), whereas an earlier analysis using a different DTI-ALPS computation pipeline found a predominantly negative coefficient; this instability in sign across pipelines means no reliable coefficient direction can be assigned. In the tree-based models, ALPS permutation importance was near zero or negative — permuting ALPS did not reduce, and on average marginally increased, held-out AUC — and ALPS ranked low among available predictors once ASL radiomic features were included (10th–13th of 14 features).

Table S11. Sensitivity of the Volume-Adjusted 365-Day Association to the Candidate Feature Pool (N=322, 90 events)

| Candidate feature pool | Candidates (n) | Volume-adjusted 365-day HR (95% CI) | p |
| --- | --- | --- | --- |
| Pre-specified GLSZM ZoneEntropy / GrayLevelNonUniformity family (primary analysis) | 26 | 1.39 (1.07–1.82) | 0.014 |
| All GLSZM features | 208 | 1.37 (1.04–1.81) | 0.027 |
| All Entropy / NonUniformity features, any texture family | 234 | 1.37 (1.05–1.79) | 0.019 |
| All non-shape ASL features (fully nested screen) | 1,209 | 1.33 (1.05–1.69) | 0.018 |

*Note. All four analyses use identical nesting: within each training fold the ten candidates with the strongest training-fold univariate Cox association were selected, and imputation and standardization used training-fold statistics only. The rows differ solely in the pool from which those candidates were drawn; each pool is defined by feature nomenclature alone, without reference to the outcome. The association is essentially unchanged across pools, including the fully nested screen in which the candidate pool itself is chosen inside the folds and no aspect of feature selection uses the full cohort. The early-mortality association therefore does not depend on the pre-specified 26-feature family. The pre-specified family is shown at 5 repeats of 5-fold cross-validation for comparability with the wider pools; at 20 repeats, as used for the primary analysis, it gives 1.39 (1.07–1.81), p=0.015.*

# Figure S1. Kaplan-Meier Survival by ASL Heterogeneity and Contralateral DTI-ALPS Phenotype

*Kaplan-Meier survival by ASL heterogeneity and contralateral DTI-ALPS phenotype, strict contralateral cohort (N=104). Patients were stratified into four groups by ASL heterogeneity (median split) and contralateral ALPS status (mean split). The unadjusted log-rank test showed a non-significant difference across groups (p=0.104). High-heterogeneity group membership correlated strongly with tumor volume (median 92.2 cm³ vs. 30.5 cm³ in the low-heterogeneity group; AUC=0.851), and the graded survival pattern did not remain significant after volume-residual regrouping (p=0.443) or volume-adjusted Cox likelihood-ratio testing (p=0.488). This phenotype analysis should be interpreted as exploratory and substantially attributable to tumor volume rather than a distinct heterogeneity–ALPS interaction.*

# Figure S2. Permutation-Based Feature Importance, ASL-Only Random Forest Classifier

Permutation-based feature importance of ASL radiomics in the ASL-only random forest classifier, with 95% confidence intervals across cross-validation folds

**Table S12. Volume-Confounding Sensitivity Analyses (Primary Cohort, N=322, 90 Deaths Within 365 Days)**

All models are volume-adjusted Cox proportional hazards models of 365-day mortality using the leakage-free out-of-fold ASL heterogeneity score, adjusted for age, sex, MGMT promoter methylation status, and extent of resection. Only the tumor-volume specification differs between rows.

**S12a. Flexible modelling of tumor volume**

| **Volume specification** | **Volume terms** | **Score HR (95% CI)** | **p** |
| --- | --- | --- | --- |
| No volume adjustment | 0 | 1.43 (1.20–1.71) | <0.001 |
| Raw linear volume | 1 | 1.47 (1.09–1.97) | 0.012 |
| Log volume, linear (primary analysis) | 1 | 1.39 (1.07–1.81) | 0.015 |
| Log volume + quadratic | 2 | 1.45 (1.08–1.93) | 0.013 |
| Log volume + quadratic + cubic | 3 | 1.76 (1.34–2.29) | <0.001 |
| Restricted cubic spline, 3 knots (log volume) | 2 | 1.44 (1.08–1.92) | 0.013 |
| Restricted cubic spline, 4 knots (log volume) | 3 | 1.45 (1.08–1.95) | 0.013 |
| Restricted cubic spline, 5 knots (log volume) | 4 | 1.49 (1.10–2.01) | 0.011 |
| Volume quintile indicators | 4 | 1.40 (1.09–1.80) | 0.009 |
| Volume decile indicators | 9 | 1.43 (1.10–1.85) | 0.008 |
| Restricted cubic spline, 5 knots (raw volume) | 4 | 1.21 (0.84–1.74) | 0.318 |

Note. Every specification using a log-transformed or non-parametric representation of volume gives a hazard ratio between 1.40 and 1.49 (p<0.02). The single exception is a five-knot restricted cubic spline fitted to untransformed volume, which spans 6,000–308,000 mm³; this design is ill-conditioned (condition number 255, versus 41 on the log scale) and the fit is unstable, as adding a small ridge penalty (0.01) moves the same estimate to 1.46 (p=0.011). The attenuation in that row therefore reflects numerical instability of an extreme specification rather than residual volume confounding.

**S12b. Stability of the score coefficient across volume strata**

| **Analysis** | **Score HR** | **p** |
| --- | --- | --- |
| Cox stratified on volume tertiles | 1.43 | 0.003 |
| Cox stratified on volume quartiles | 1.37 | 0.016 |
| Cox stratified on volume quintiles | 1.41 | 0.009 |
| Within volume tertile 1 (smallest tumors) | 1.25 | 0.41 |
| Within volume tertile 2 | 1.23 | 0.24 |
| Within volume tertile 3 (largest tumors) | 1.38 | 0.014 |
| Fixed-effect estimate pooled across tertiles | 1.32 (95% CI 1.09–1.59) | — |
| Score × log-volume interaction term | 0.99 (95% CI 0.80–1.23) | 0.927 |

Note. Stratified models allow a separate baseline hazard within each volume stratum. Between-stratum heterogeneity of the score coefficient was absent (Cochran Q = 0.32, df = 2, p = 0.853; I² = 0.0%), and the score-by-volume interaction was null, indicating the effect does not vary with tumor size. Each tertile contains approximately one third of the cohort, so the within-stratum analyses are individually underpowered and their wide confidence intervals should be interpreted accordingly.

**S12c. Orthogonalization of the heterogeneity score with respect to volume**

| **Residualization of score on tumor volume** | **Spearman ρ (residual, volume)** | **Score HR (95% CI)** | **p** |
| --- | --- | --- | --- |
| Linear, on log volume | −0.016 | 1.25 (1.05–1.50) | 0.013 |
| Quadratic + cubic, on log volume | −0.109 | 1.26 (1.04–1.53) | 0.019 |
| Restricted cubic spline, 4 knots | −0.110 | 1.26 (1.04–1.52) | 0.018 |
| Restricted cubic spline, 5 knots | −0.094 | 1.27 (1.05–1.54) | 0.015 |
| Rank-based (Spearman scale) | +0.049 | 1.27 (1.02–1.58) | 0.030 |
| Spline, 5 knots, with volume retained in the model | −0.094 | 1.25 (1.05–1.49) | 0.010 |

Note. The residualized score is uncorrelated with tumor volume by construction, so an association that persists after residualization cannot be carried by volume. The orthogonalized hazard ratios (1.25–1.27) are smaller than the primary estimate of 1.39, indicating that part of the score's prognostic signal is shared with tumor volume while a volume-independent component remains.

**S12d. Negative-control calibration (2,000 replicates per configuration)**

| **Null model** | **Volume adjustment** | **Type-I error at α=0.05** | **Median null HR** | **95th percentile null HR** | **Empirical p for observed HR** |
| --- | --- | --- | --- | --- | --- |
| Gaussian copula, matched to observed score–volume ρ | Log volume | 3.90% | 1.00 | 1.34 | 0.035 |
| Gaussian copula, matched to observed score–volume ρ | Spline, 5 knots | 5.05% | 0.99 | 1.40 | 0.052 |
| Permutation of the observed score within volume deciles | Log volume | 4.20% | 0.99 | 1.25 | 0.008 |
| Permutation of the observed score within volume deciles | Spline, 5 knots | 5.30% | 0.98 | 1.27 | 0.014 |

Note. Both null models construct variables that carry the heterogeneity score's relationship to tumor volume but no independent prognostic content. The permutation null preserves the observed score–volume relationship most closely, because it reuses the observed score values and only exchanges them among patients of near-identical tumor volume. Type-I error at or near the nominal 5% in every configuration indicates that volume adjustment is calibrated rather than anticonservative, and the empirical p values indicate that a pure volume proxy rarely reproduces the observed hazard ratio. One configuration, the smoothest synthetic null tested against the most flexible adjustment, was borderline (empirical p=0.052) and is reported as obtained.

**Table S13. Inverse-Probability-of-Censoring-Weighted Sensitivity Analysis (365-Day Endpoint, N=322)**

| **Model** | **N** | **Deaths ≤365 days** | **Score HR (95% CI)** | **p** |
| --- | --- | --- | --- | --- |
| Unweighted (primary analysis) | 322 | 90 | 1.39 (1.07–1.81) | 0.015 |
| Inverse-probability-of-censoring weighted | 322 | 90 | 1.40 (1.14–1.78)* | ≈0.01 |
| Complete-case (patients censored <365 days excluded) | 259 | 90 | 1.38 (1.07–1.79) | 0.013 |

Note. Sixty-three of 322 patients were censored before 365 days and were older than those retained (Supplementary Table S8). Stabilized inverse-probability-of-censoring weights were estimated from age, sex, MGMT promoter methylation status, extent of resection, and log tumor volume (mean weight 1.005, range 0.887–1.242), and truncated at the 1st and 99th percentiles. *Confidence interval obtained by patient-level bootstrap (500 replicates); the model-based sandwich interval is not reported because weighted-variance estimation was unstable at these near-unit weights. The weighted and complete-case estimates are both essentially identical to the primary estimate, indicating that censoring before 365 days did not materially bias the primary analysis.
